# Piperidine-Iodine as Efficient Dual Catalyst for the One-Pot, Three-Component Synthesis of Coumarin-3-Carboxamides

**DOI:** 10.3390/molecules27144659

**Published:** 2022-07-21

**Authors:** Manuel Velasco, Nancy Romero-Ceronio, Rosalía Torralba, Oswaldo Hernández Abreu, Miguel A. Vilchis-Reyes, Erika Alarcón-Matus, Erika M. Ramos-Rivera, David M. Aparicio, Jacqueline Jiménez, Eric Aguilar García, David Cruz Cruz, Clarisa Villegas Gómez, Cuauhtémoc Alvarado

**Affiliations:** 1 División Académica de Ciencias Básicas, Universidad Juárez Autónoma de Tabasco, Carretera Cunduacán-Jalpa Km 1, Col. La Esperanza, Cunduacán C.P. 86690, Tabasco, Mexico; manuel.velasco@ujat.mx (M.V.); nancy.romero@ujat.mx (N.R.-C.); oswaldo.hernandez@ujat.mx (O.H.A.); miguel.vilchis@ujat.mx (M.A.V.-R.); erika.alarcon@ujat.mx (E.A.-M.); erika.ramos@ujat.mx (E.M.R.-R.); 2 Complejo Regional Mixteca, Campus Izúcar de Matamoros, Benemérita Universidad Autónoma de Puebla, Carr. Atlixco-Izúcar de Matamoros 141, San Martín Alchichica, Izúcar de Matamoros C.P. 74570, Puebla, Mexico; rosalia.torralbasan@correo.buap.mx; 3 Instituto de Ciencias, Benemérita Universidad Autónoma de Puebla, Edif. IC-9 Complejo de Ciencias C.U. 72570, Puebla, Mexico; davidmiguel.aparicio@correo.buap.mx; 4 Facultad de Ciencias Químicas, Benemérita Universidad Autónoma de Puebla, Puebla C.U. 72570, Puebla, Mexico; jacqueline.jimenez@correo.buap.mx (J.J.); eric.aguilargarcia@gmail.com (E.A.G.); 5 Departamento de Química, División de Ciencias Naturalesy Exactas, Universidad de Guanajuato, Noria Alta S/N 36050, Guanajuato, Mexico; david.cruz@ugto.mx (D.C.C.); clarisa.villegas@ugto.mx (C.V.G.)

**Keywords:** multicomponent reaction, molecular iodine, coumarin-3-carboxamides

## Abstract

A simple and efficient one-pot, three-component synthetic method for the preparation of coumarin-3-carboxamides was carried out by the reaction of salicylaldehyde, aliphatic primary/secondary amines, and diethylmalonate. The protocol employs piperidine-iodine as a dual system catalyst and ethanol, a green solvent. The main advantages of this approach are that it is a metal-free and clean reaction, has low catalyst loading, and requires no tedious workup.

## 1. Introduction

Today, the development of efficient and environmentally friendly synthesis to obtain complex and highly substituted molecules is one of the most exciting topics for the synthetic chemistry community. In this sense, multicomponent reactions (MCRs) have emerged as a powerful synthetic tool in organic synthesis as an attractive alternative to the conventional multi-step synthesis. These multicomponent strategies provide great molecular diversity in a single step and in a highly efficient manner [1,2,3]. The utility of these processes has been confirmed by the synthesis of a large number of compounds with remarkable biological activity [4,5].

Among the pharmacologically active products, coumarin and its derivatives have attracted attention due to their broad spectrum of applications in medicinal chemistry [6,7]. In particular, coumarin-3-carboxamide has proven to be an important structural core that exhibits diverse biological activities, such as anticancer [8,9], antioxidant [10,11], anti-inflammatory [12], and anticoagulant [13] properties, as well as inhibition against β-secretase (BACE1) [14,15], monoamine oxidase (MAO) [16], acetylcholinesterase [17], and tumorigenesis [18]. In addition, these compounds have found applications as fluorescent probes for Cu^2+^ and Fe^3+^ detection [19,20] and molecular sensors for monitoring O_2_ levels in living cells [21].

**Figure 1:**
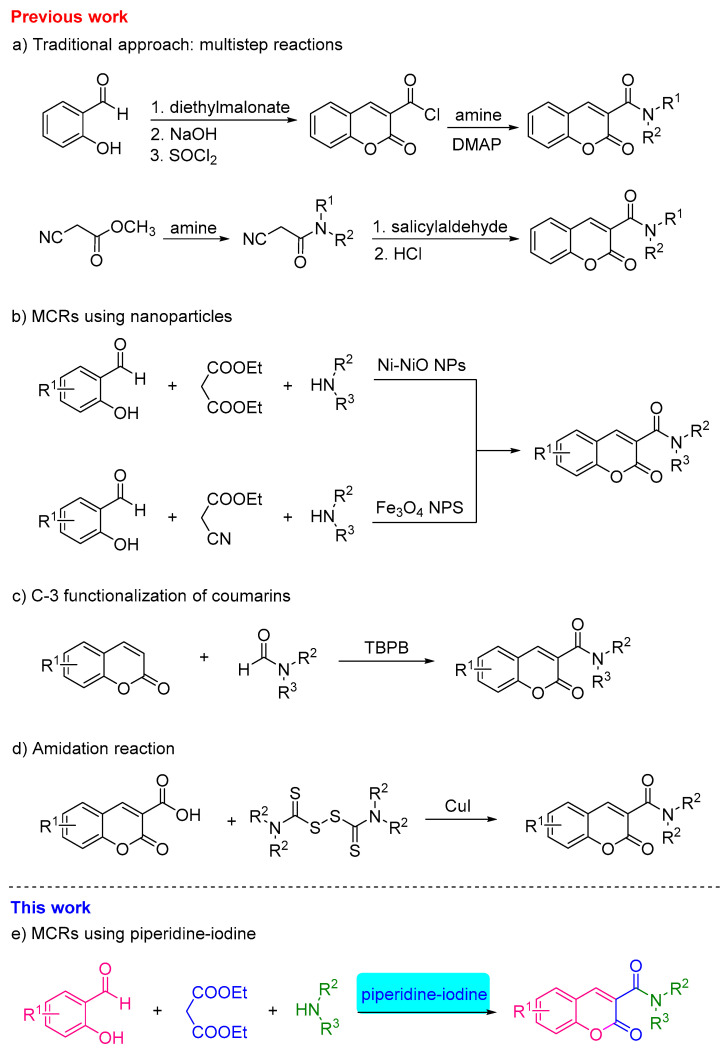
General methods for the synthesis of coumarin-3-carboxamides: (**a**) Traditional approach multistep reactions [22], (**b**) MCRs using nanoparticles [24,25], (**c**) C-3 functionalization of coumarins [26], (**d**) amidation reaction [27], (**e**) MCRs using piperidine-iodine.

Accordingly, several strategies for the synthesis of this important class of compounds have been described in the literature. The traditional approach involves multistep elementary reactions including condensation reactions between activated coumarin derivatives or methyl cyanoacetate with amines (Figure 1a) [22,23]. Alternatively, MCRs using magnetic nanoparticles (NPs) such as Ni–NiO and Fe_3_O_4_ have also been applied in the synthesis of a variety of coumarin-3-carboxamides (Figure 1b) [24,25]. Other reported approaches to the synthesis of coumarin-3-carboxamide involve regioselective carboxamidation of coumarins at C-3 with formamides by using the radical initiator *tert*-butyl peroxybenzoate (TBPB) (Figure 1c) [26], and amidation reactions of coumarin-3-carboxylic acids using tetraalkylthiuram disulfides as amine sources (Figure 1d) [27].

However, most of the above-mentioned protocols suffer from several limitations such as pore functionalization of the substrates, harsh reaction conditions, expensive reagents, long reaction times, laborious workup, and metal-based catalysis. Therefore, from the environmental and economic perspectives, the development of an easy-to-implement protocol for the preparation of coumarin-3-carboxamides would be of great importance in synthetic organic chemistry.

On the other hand, iodine-catalyzed reactions have attracted great research interest because of their simplicity in operation, enhanced reaction rates, and great selectivity. Iodine is also a cheap, non-toxic, and water-tolerant catalyst [28,29,30], which has been explored as a powerful catalyst for MCRs, due to its unique catalytic properties [31,32,33,34].

Based on the use of iodine and the fact that, to the best of our knowledge, no free metal and one-pot syntheses of coumarin-3-carboxamides have been reported, we present here a simple and efficient one-pot, three-component synthetic method for the preparation of coumarin-3-carboxamides using piperidine and molecular iodine as a dual-catalyst system (Figure 1e). The notable advantages of this protocol are (a) transition-metal-free protocol, (b) use of iodine as a catalyst, (c) use of ethanol as a solvent, and (d) workup simplicity. 

## 2. Results

**Scheme 1:**
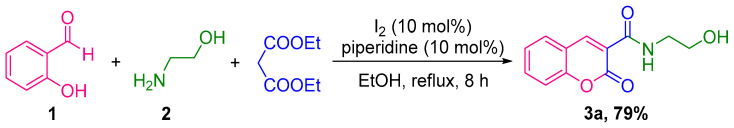
Multicomponent reaction of 2-hydroxybenzaldehyde, ethanolamine, and diethyl malonate.

Initially, we explore the multicomponent reaction involving 2-hydroxybenzaldehyde (**1**), ethanolamine (**2**), and diethyl malonate (DEM). The reaction was carried out in reflux ethanol in the presence of iodine (10 mol%) and piperidine (10 mol%) over the course of 8 h. As a result, the 3-amidocoumarin **3a** was afforded at a 79% yield (Scheme 1).

In view of this success, the optimization of the reaction was performed. Thus, the effects of the solvent, base, and catalyst were studied (Table 1). The solvent effect was studied in the first place, and in a general way, protic solvents produced superior results to aprotic solvents in terms of selectivity (Table 1, entries 1–4 vs. 6–10), since the coumarin-3-carboxamide **3a** was the only product detected, though in regular yields (37–79%). Among the protic solvents, the green solvent ethanol [35] was the most effective (Table 1, entry 1), because compound **3a** was obtained with the best yield (79%). Otherwise, the use of water and aprotic polar solvents such as CH_2_Cl_2_, MeCN, EtOAc, THF, and DMF provided a mixture of coumarin-3-carboxamide **3a**, imine **4a,** and ethyl coumarin-3-carboxylate **5** (Table 1, entries 5–10). The solvent-free synthesis was found to be interesting (Table 1, entry 11); unfortunately, it required a high temperature (250 °C) and up to 14 h to complete the reaction. In order to establish the role of piperidine, different bases such as Et_3_N, 1,8-diazabicycloundec-7-ene (DBU), and L-proline were tested, and none of them were effective for the obtention of compound **3a** (Table 1, entries 12−14), sadly. Finally, the ratio of base to iodine was investigated (Table 1, entries 15−21). We observed that increasing the amount of iodine from 10 mol% to 20 mol% resulted in a slight decrease in the reaction yield from 79% to 75% (Table 1, entry 1 vs. entry 15). The use of 15 mol% did not affect the product yield (Table 1, entry 16). Fortunately, when using 5 mol% of iodine, the highest yield (85%) for the reaction was found (Table 1, entry 17). However, on reducing the amount of iodine, the product yield decreased (Table 1, entry 18).

**Scheme 2:**
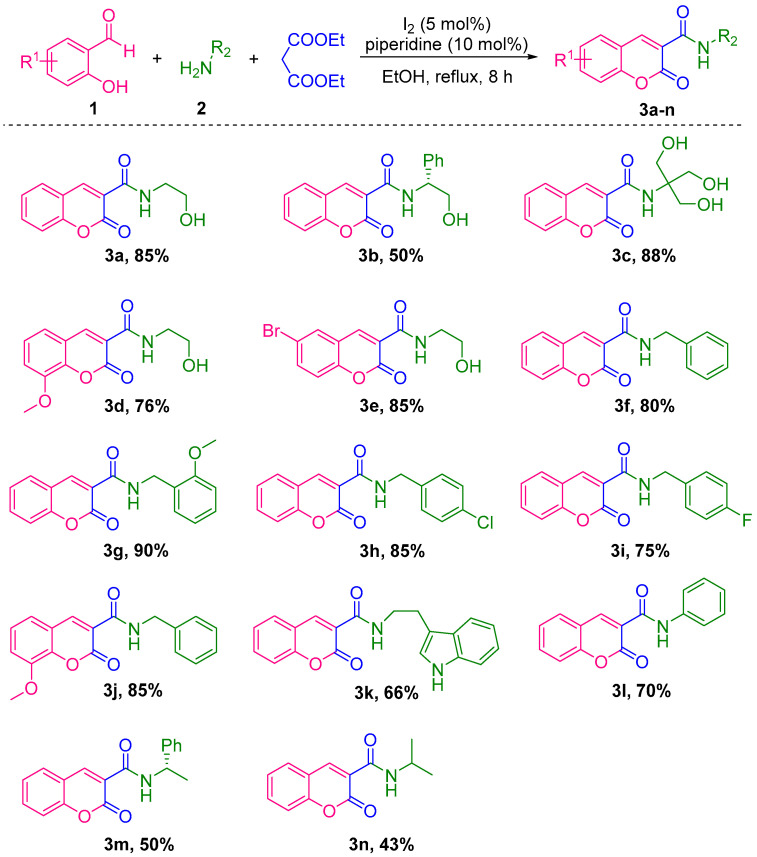
Substrate scope for the multicomponent reaction of salicylaldehyde, primary amines, and DEM.

**Scheme 3:**
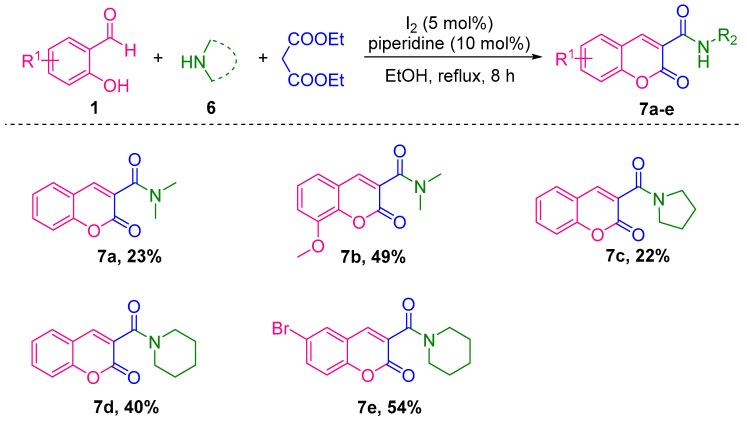
Substrate scope for the multicomponent reaction of salicylaldehyde, secondary amine, and DEM.

Having established the optimal reaction conditions, we subsequently explored the scope for the synthesis of various coumarin-3-carboxamides **3a–n** and **7a–e** (Scheme 2 and Scheme 3) for this methodology. Accordingly, differently functionalized 2-hydroxy-benzaldehydes **1** were subjected to the multicomponent reaction with diverse aminoalcohols, primary amines **2,** and DEM (Scheme 2). Starting with aminoalcohols, the results showed good to very good yields of products **3a**, **3d,** and **3e** (85%, 76%, and 85%, respectively) when ethanolamine, a primary, unhindered amine was used. The yield diminished (**3b**, 50%) when (*R*)-(–)-2-phenylglycinol, a hindered amine was used, and surprisingly, the yield obtained using 2-amino-2-(hydroxymethyl)propane-1,3-diol was very good (**3c**, 88%). This result may be due to the formation of a hydrogen bond between hydroxyl groups of 2-amino-2-(hydroxymethyl)propane-1,3-diol and the carbonyl group of DEM (SI, Scheme S1). Primary amines attached to methylene carbons (i.e., benzylamine and tryptamine) reacted satisfactorily, and the corresponding products **3f**–**k** were obtained with regular to very good yields (66–90%). The coumarin-3-carboxamide **3l**, derived from aniline (an aromatic amine) was isolated in regular yield (70%). As expected, yields diminished when the primary amine was attached to a methine carbon, due to the increase in steric hindrance. For example, (*S*)-(–)-α-methylbenzylamine produced **3m** at a 50% yield, while isopropylamine delivered **3n** in 43% yield. Additionally, the influence of substituents on the aromatic ring of aldehydes was tested. A soft decrease in yield (**3d**, 76%) was observed when using 3-methoxysalicylaldehyde (with an electron-releasing group). In contrast, a very good yield (**3e**, 85%) was obtained when using 5-bromosalicylaldehyde (with an electron-withdrawing group).

To further explore the substrate scope, we investigated the multicomponent reaction with secondary amines (including cyclic and aliphatic) **6** (Scheme 3). Unfortunately, yields were from low to regular (22%–54%); for instance, a 23% yield of **7a** was obtained when using dimethylamine, and only a 54% yield of compound **7e** was isolated when using piperidine, a cyclic amine. Indeed, no reaction occurred when diethylamine, diisopropylamine, or diphenylamine was used (not shown). Again, some unfavorable steric factors may be responsible for both the low yield and not the formation of coumarin-3-carboxamides from long chain secondary amines [24].

Structures of synthesized compounds were confirmed by analytical and spectral data (see SI). In a representative example, the ^1^H-NMR spectrum of *N*-(2-hydroxyethyl)-2-oxo-2*H*-chromene-3-carboxamide (**3a**) showed one singlet signal at δ = 8.90 ppm, corresponding to H-4 (alkene) of the coumarin nucleus. Signals at 8.00, 7.76, 7.52, and 7.45 ppm were assigned to aromatic protons of the coumarin core. NH and OH protons appear as triplet signals at δ = 8.86 and 4.92 ppm, respectively. Two quadruplet signals at δ = 3.55 and 3.41 ppm were assigned to the protons of the two methylene. The ^13^C NMR spectrum of **3a** exhibited 12 distinct signals in agreement with the suggested structure [36].

**Scheme 4:**
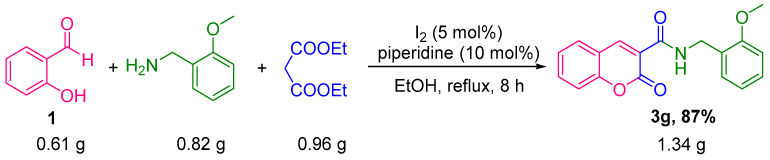
Synthesis of **3g** at a 5 mmol scale.

In order to show the applicability of this methodology, compound **3g** was synthesized at a 5 mmol scale. As a result, compound **3g** was afforded at a 87% yield (1.34 g). This result further proved the feasibility to apply this methodology to a larger-scale process (Scheme 4).

**Scheme 5:**
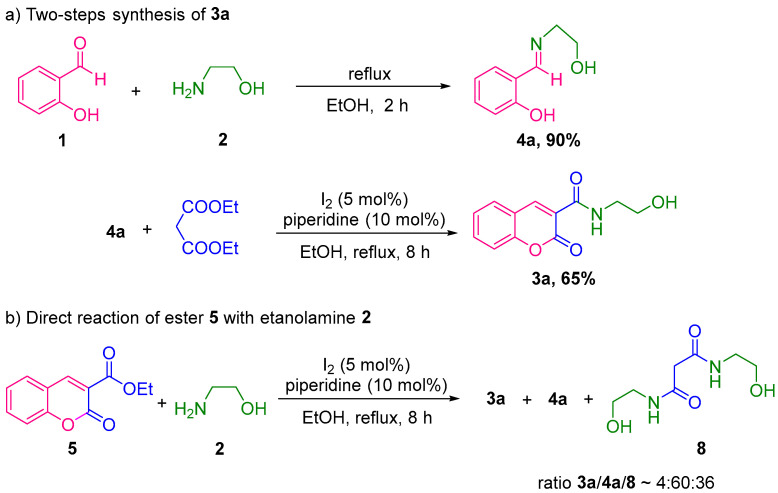
Control experiments.

To gain insight into the reaction mechanism, control experiments were conducted as shown in Scheme 5. Initially, 2-hydroxybenzaldehyde (**1**) and ethanolamine (**2**) were stirred in ethanol at reflux for 2 h, and the imine **4a** could be isolated at a 90% yield. Then, imine **4a** was further reacted with DEM under standard conditions; this way, the target product **3a** could be obtained at a 65% yield (Scheme 5a). Next, the ester **5** was employed in the direct reaction with ethanolamine, and under the standard conditions, a mixture of the 3-amidocoumarin **3a**, imine **4a,** and *N^1^,N^3^-*bis(2-hydroxyethyl)malonamide **8** (4:60:36) was obtained (Scheme 5b). The obtained result indicates that the formation of **3a** from the coumarin **5** is not efficient, mainly due to a competitive reaction involving the attack at the 4-position of coumarin [37] and also due to the fact that imine **4a** is the most likely intermediate of the reaction.

**Scheme 6:**
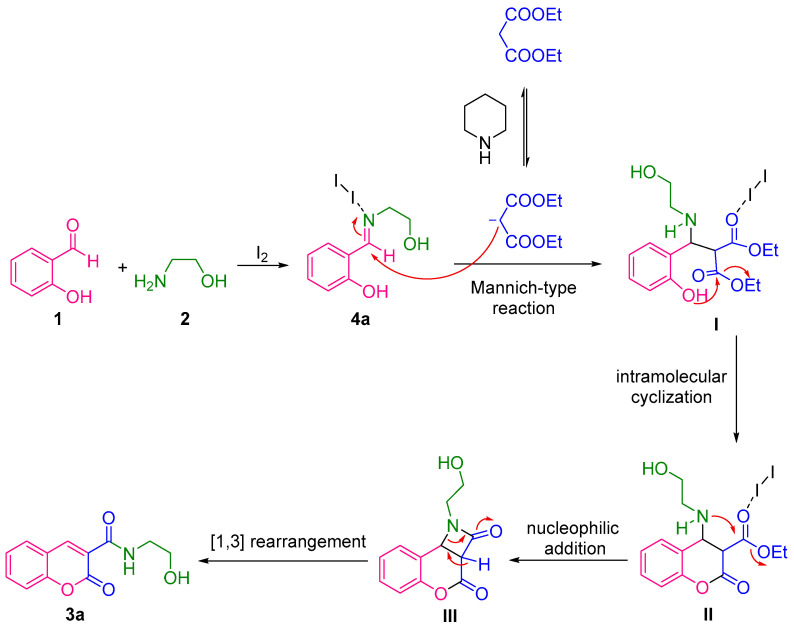
Plausible reaction pathway.

Based on the above results, a plausible mechanism is proposed (Scheme 6). Firstly, the condensation between **1** and ethanolamine **2**, in the presence of iodine, leads to imine **4a**. Secondly, imine **4a** undergoes a Mannich-type reaction with DEM in the presence of piperidine to form intermediate **I**. Thirdly, an intramolecular cyclization produces intermediate chroman-2-one **II**, which suffers a second intramolecular cyclization to afford intermediate beta-lactam **III**. Finally, a [1,3]-amino rearrangement involving the β-lactam results in the formation of the target molecule **3a** [38]. During the reaction mechanism, iodine is proposed to activate the imine and carbonyl groups as it behaves as a mild Lewis acid [39], thus facilitating the transformations, as long as piperidine acts as a base, which allows for protonic transferences [32].

## 3. Materials and Methods

### 3.1. General Information

All chemicals were purchased from Sigma Aldrich (Toluca, Mexico). Melting points were determined on a Stuart SMP10 apparatus by the open capillary technique and are uncorrected. ^1^H and ^13^C NMR spectra were recorded at 600 MHz and 150 MHz, respectively, in CDCl_3_ or DMSO-d6 using a Bruker Ascend^TM^ Spectrometer. Chemical shifts are given in ppm and reported to the residual solvent peak (CDCl_3_: 7.26 ppm for ^1^H and 77.16 ppm for ^13^C; DMSO-*d*_6_: 2.50 ppm for ^1^H and 39.51 ppm for ^13^C). Data are reported as follows: chemical shift (δ), multiplicity (s = singlet, d = doublet, t = triplet, q = quartet, m = multiplet, br s = broad singlet), coupling constant(s) (*J*, Hz), and integration. Analytical TLC was performed on silica gel 60 F_254_ plates. IR spectra were obtained using an FT-IR spectrometer, Spectrum One, Perkin Elmer. 

### 3.2. General Procedures and Compound Characterization Data for Coumarin-3-carboxamides ***3a–n*** and ***7a–e***

To a stirred solution of salicylaldehyde (0.122 g, 1 mmol), primary or secondary amine (1.2 mmol) and diethyl malonate (1.2 mmol) in absolute ethanol (2 mL) was treated with piperidine (10 mol%) and iodine (5 mol%) and refluxed for 8h. After completion, the mixture was filtered and the precipitate was washed with cold ethanol (4 mL) to afford the pure products **3a**–**n** and **7a**–**e**. If necessary, further purification was performed by recrystallization from ethanol. The identity of the known products was confirmed by a comparison of their spectroscopic data and physical properties [20,24,40,41,42,43,44,45,46]. The characterization data of synthesized compounds are given in the Supplementary Materials. 

## 4. Conclusions

We have developed a piperidine/iodine-promoted three-component reaction for the synthesis of various coumarin-3-carboxamides. The simplicity of the synthetic protocol and availability of diverse starting materials make this an attractive strategy for the synthesis of this class of compounds. Further applications of this catalytic system to other multicomponent reactions are now underway.

## Figures and Tables

**Table 1:** Optimization of the reaction conditions ^a^.

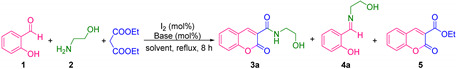					
Entry	Solvent	Base (mol%)	Iodine (mol%)	Ratio ^b^ (3a:4a:5)	Yield 3a ^c^ (%)
1	EtOH	piperidine 10%	10	100:0:0	79
2	MeOH	piperidine 10%	10	100:0:0	50
3	*i*-PrOH	piperidine 10%	10	100:0:0	38
4	*t*-BuOH	piperidine 10%	10	100:0:0	37
5	H_2_O	piperidine 10%	10	35:55:10	14
6	CH_2_Cl_2_	piperidine 10%	10	28:2:70	8
7	MeCN	piperidine 10%	10	75:0:25	32
8	EtOAc	piperidine 10%	10	51:14:35	26
9	THF	piperidine 10%	10	66:24:10	17
10	DMF	piperidine 10%	10	21:53:26	10
11	Solvent free	piperidine 10%	10	100:0:0	36
12	EtOH	Et_3_N 10%	10	100:0:0	40
13	EtOH	DBU 10%	10	100:0:0	49
14	EtOH	L-Proline 10%	10	100:0:0	35
15	EtOH	piperidine 10%	20	100:0:0	75
16	EtOH	piperidine 10%	15	100:0:0	79
17	EtOH	piperidine 10%	5	100:0:0	85
18	EtOH	piperidine 10%	1	100:0:0	60
19	EtOH	piperidine 5%	5	100:0:0	72
20	EtOH	-	5	100:0:0	63
21	EtOH	piperidine 10%	-	100:0:0	66

^a^ Reactions were performed at scale of 1 mmol. Relationship salicylaldehyde:ethanolamine:DEM (1.0:1.2:1.2). ^b^ Ratios were calculated from the NMR spectrum of the crude reaction. ^c^ Isolated yields.

## Data Availability

Not applicable.

## Supplementary Materials

The following supporting information can be downloaded at: https://www.mdpi.com/article/10.3390/molecules27144659/s1, characterization data, and ^1^H-NMR and ^13^C-NMR of compounds **3a–n** and **7a–e**.

## Sample Availability

Samples of the compounds are not available from the authors.
