# Supplementary material for: Piperidine-Iodine as Efficient Dual Catalyst for the One-Pot, Three-Component Synthesis of Coumarin-3-Carboxamides
Source: Molecules. 2022 Jul 21;27(14):4659. doi: 10.3390/molecules27144659 (PMC9323834; doi:10.3390/molecules27144659)
Supplement: Supplementary file 1 [file molecules-27-04659-s001.zip › molecules-1798351-supplmentary.pdf]

Supplementary Information

Table of Contents

General Information S2

General Procedures and Characterization data for Coumarin-3-carboxamides **3a-n** and **7a-e** S2

^{1}H and ^{13}C NMR for coumarin-3-carboxamides **3a-n** and **7a-e** S10

Mechanistic details for the formation of **3c** S31

General Information:

All chemicals were purchased from Sigma Aldrich (Toluca, Mexico). Melting points were determined on a Stuart SMP10 apparatus by the open capilar technique and are uncorrected. ^{1}H and ^{13}C NMR spectra were recorded at 600 MHz and 150 MHz, respectively, in $CDCl_3$ or DMSO-d_6 using a Bruker Ascend^{TM} Spectrometer. Chemical shifts are given in ppm and reported to the residual solvent peak ($CDCl_3$: 7.26 ppm for ^{1}H and 77.16 ppm for ^{13}C; DMSO-d_6: 2.50 ppm for ^{1}H and 39.51 ppm for ^{13}C). Data are reported as follows: chemical shift (δ), multiplicity (s = singlet, d = doublet, t = triplet, q = quartet, m = multiplet, br s = broad singlet), coupling constant(s) (*J*, Hz), and integration. Analytical TLC was performed on silica gel 60 F_{254} plates. IR spectra were obtained using an FT-IR spectrometer, Spectrum One, Perkin Elmer.

General Procedures and compound Characterization Data for Coumarin-3-carboxamides 3a-n and 7a-e.

To a stirred solution of salicylaldehyde (0.122g, 1 mmol), primary or secondary amine (1.2 mmol) and diethyl malonate (1.2 mmol) in absolute ethanol (2ml) was treated with piperidine (10 mol%) and iodine (5 mol%) and refluxed for 8h. After completion, the mixture was filtered and the precipitate washed with cold ethanol (4 mL) to afford the pure products **3a-n** and **7a-e**. If necessary, further purification was performed by recrystallization from ethanol. The identity of the known products was confirmed by comparison of their spectroscopic data and physical properties [20, 24, 40-46].

***N*-(2-hydroxyethyl)-2-oxo-2*H*-chromene-3-carboxamide (3a).** As white solid; yield-85%; Mp 179-180 °C, [Lit: Mp 179-181 °C] [40]; FT-IR ν_{max}/cm^{-1} 3391, 3324, 1700, 1640, 1605;

^1H NMR (600 MHz, DMSO-d_6) δ 8.90 (s, 1H), 8.86 (t, 1H, *J* = 5.5 Hz, NH), 8.00 (dd, 1H, *J* = 7.8 and 1.6 Hz), 7.76 (ddd, 1H, *J* = 8.7, 7.3 and 1.6 Hz), 7.52 (d, 1H, *J* = 8.3 Hz), 7.45 (td, 1H, *J* = 7.6 and 1.5 Hz), 4.92 (t, 1H, *J* = 5.6 Hz, OH), 3.55 (q, 2H, *J* = 5.6 Hz), 3.41 (q, 2H, *J* = 5.6 Hz); $^{13}C\{^1H\}$-NMR (150 MHz, DMSO-d_6) δ 161.0, 160.5, 153.8, 147.6, 134.1, 130.3, 125.1, 118.7, 118.4, 116.1, 59.4, 41.8;

(*R*)-*N*-(2-hydroxy-1-phenylethyl)-2-oxo-2*H*-chromene-3-carboxamide (3b). As light yellow solid; yield- 50%; Mp 159-161 °C; FT-IR ν_{max}/cm^{-1} 3339, 3298, 1710, 1644, 1600, 1542; $[\alpha]_D^{20}$= +16.5 (*c* 1.0, $CHCl_3$); ^1H NMR (600 MHz, $CDCl_3$) δ 9.48 (d, 1H, *J* = 7.4 Hz, NH), 8.82 (s, 1H), 7.60 (ddd, 2H, *J* = 14.5, 8.3 and 1.5 Hz), 7.33-7.29 (m, 6H), 7.24-7.21 (m, 1H), 5.24 (dd, 1H, *J* = 11.7 and 6.6 Hz), 3.90-3.89 (m, 2H), 2.72 (br s, 1H, OH); $^{13}C\{^1H\}$-NMR (150 MHz, $CDCl_3$) δ 162.0, 161.5, 154.5, 148.7, 138.6, 134.3, 129.9, 129.0, 128.0, 126.8, 125.4, 118.6, 118.2, 116.7, 67.0, 56.6.

***N*-(1,3-dihydroxy-2-(hydroxymethyl)propan-2-yl)-2-oxo-2*H*-chromene-3-carboxamide (3c).** As white solid; yield- 88%; Mp 208-210 °C, [Lit: Mp 209-211 °C] [20]; FT-IR ν_{max}/cm^{-1} 3385, 3333, 3278, 1698, 1643, 1608, 1554, 1042, 776; ^1H NMR (600 MHz, DMSO-d_6) δ 8.99 (s, 1H, NH), 8.92 (s, 1H), 7.99 (dd, 1H, *J* = 7.8 and 1.6 Hz), 7.77 (ddd, 1H, *J* = 8.7, 7.3 and 1.6 Hz), 7.53 (d, 1H, *J* = 8.3 Hz), 7.46 (td, 1H, *J* = 7.5 and 1.1 Hz), 4.86 (t, 3H, *J* = 5.6 Hz, OH), 3.69 (d, 6H, *J* = 5.6 Hz); $^{13}C\{^1H\}$-NMR (150 MHz, DMSO-d_6) δ 160.9, 160.6, 153.8, 147.6, 134.1, 130.2, 125.1, 118.8, 118.3, 116.0, 62.3, 60.0.

***N*-(2-hydroxyethyl)-8-methoxy-2-oxo-2*H*-chromene-3-carboxamide (3d).** As white solid; yield-76%; Mp 198-200 °C, [Lit: Mp 194-196 °C] [41] ; FT-IR ν_{max}/cm^{-1} 3371, 3310, 1706, 1634, 1603, 1277, 794; ^1H NMR (600 MHz, DMSO-d_6) δ 8.85 (s, 2H), 7.52 (dd, 1H,

J = 7.38 and 1.4 Hz), 7.43 (dd, 1H, *J* = 8.3 and 1.4 Hz), 7.37 (t, 1H, *J* = 7.9), 4.90 (t, 1H, *J* = 5.1 Hz, OH), 3.94 (s, 3H), 3.55 (q, 2H, *J* = 5.5 Hz), 3.42 (t, 2H, *J* = 5.6 Hz); $^{13}C\{^{1}H\}$-NMR (150 MHz, DMSO-d_6) δ 161.5, 160.7, 148.3, 146.7, 143.6, 125.6, 121.6, 119.5, 119.3, 116.5, 59.9, 56.6, 42.3.

6-bromo-*N*-(2-hydroxyethyl)-2-oxo-2*H*-chromene-3-carboxamide (3e). As white solid; yield-85%; Mp 204-206 °C, [Lit : Mp 204 °C] [42]; FT-IR $ν_{max}/cm^{-1}$ 3473, 3343, 3052, 1712, 1657, 1611, 1556, 1068, 846; ^{1}H NMR (600 MHz, DMSO-d_6) δ 8.85 (s, 1H), 8.81 (t, 1H, *J* = 5.6 Hz, NH), 8.26 (d, 1H, *J* = 2.4 Hz), 7.89 (dd, 1H, *J* = 8.9 and 2.4 Hz), 7.48 (d, 1H, *J* = 8.8 Hz), 4.89 (t, 1H, *J* = 5.1 Hz, OH), 3.54 (q, 2H, *J* = 5.4 Hz), 3.41 (q, 2H, *J* = 5.4 Hz); $^{13}C\{^{1}H\}$-NMR (150 MHz, DMSO-d_6) δ 161.2, 160.5, 153.4, 146.7, 136.7, 132.6, 120.8, 120.3, 118.9, 117.1, 59.9, 42.4.

***N*-benzyl-2-oxo-2*H*-chromene-3-carboxamide (3f).** As colorless crystalline solid; yield-85%; Mp 159-160 °C, [Lit : Mp 160-162 °C] [24]; FT-IR $ν_{max}/cm^{-1}$ 3330, 3310, 3278, 1702, 1654, 1611, 1565, 1244, 769; ^{1}H NMR (600 MHz, $CDCl_3$) δ 9.10 (br s, 1H, NH), 8.88 (s, 1H), 7.63 (dd, 1H, *J* = 7.8 and 1.6 Hz), 7.59 (ddd, 1H, *J* = 8.7, 7.4 and 1.6 Hz), 7.34-7.26 (m, 6H), 7.22-7.19 (m, 1H), 4.60 (d, 2H, *J* = 5.8 Hz); $^{13}C\{^{1}H\}$-NMR (150 MHz, $CDCl_3$) δ 161.6, 161.4, 154.5, 148.6, 137.9, 134.1, 129.8, 128.7, 127.7, 127.5, 125.3, 118.7, 118.4, 116.7, 43.9.

***N*-(2-methoxybenzyl)-2-oxo-*2H*-chromene-3-carboxamide (3g).** As white crystals; yield-90%; Mp 184-186 °C, [Lit : Mp 176.5-177.8 °C] [43]; FT-IR $ν_{max}/cm^{-1}$ 3369, 3032, 1700, 1657, 1607, 1562, 1241, 749; ^{1}H NMR (600 MHz, $CDCl_3$) δ 9.27 (br s, 1H, NH), 8.84 (s, 1H), 7.61 (dd, 1H, *J* = 7.7 and 1.6 Hz), 7.57 (ddd, 1H, *J* = 8.7, 7.3 and 1.6 Hz), 7.32-7.25 (m,

3H), 7.20 (td, 1H, *J* = 7.8 and 1.7 Hz), 6.86-6.82 (m, 2H), 4.59 (d, 2H, *J* = 5.9 Hz), 3.85 (s, 3H); $^{13}C\{^1H\}$-NMR (150 MHz, $CDCl_3$) δ 161.3, 161.2, 157.7, 154.4, 148.3, 133.9, 129.8, 129.5, 128.9, 126.0, 125.2, 120.6, 118.8, 118.7, 116.6, 110.4, 55.4, 39.8.

***N*-(4-chlorobenzyl)-2-oxo-*2H*-chromene-3-carboxamide (3h).** As colorless crystalline solid; yield-85%; Mp 178-180 °C, [Lit: Mp 176-178 °C] [24]; FT-IR ν_{max}/cm^{-1} 3336, 3050, 2940, 1700, 1655, 1607, 1563, 1243, 761, 530; ^1H NMR (600 MHz, $CDCl_3$) δ 9.12 (br s, 1H, NH), 8.87 (s, 1H), 7.63 (dd, 1H, *J* = 7.7 and 1.6 Hz), 7.60 (ddd, 1H, *J* = 8.7, 7.3 and 1.6 Hz), 7.34-7.30 (m, 2H), 7.24-7.21 (m, 4H), 4.55 (d, 2H, *J* = 6.0 Hz); $^{13}C\{^1H\}$-NMR (150 MHz, $CDCl_3$) δ 161.7, 161.5, 154.5, 148.8, 136.5, 134.2, 133.3, 129.9, 129.1, 128.8, 125.4, 118.6, 118.3, 116.7, 43.2.

***N*-(4-fluorobenzyl)-2-oxo-*2H*-chromene-3-carboxamide (3i).** As white solid; yield yield-75%; Mp 178-180 °C, [Lit: Mp 174.4-175.6 °C] [43]; FT-IR ν_{max}/cm^{-1} 3328, 3049, 2940, 1701, 1655, 1608, 1563, 1506, 1213, 1157, 760, 555; ^1H NMR (600 MHz, DMSO-d_6) δ 9.15 (t, 1H, *J* = 6.1 Hz, NH), 8.87 (s, 1H), 7.98 (dd, 1H, *J* = 7.8 and 1.6 Hz), 7.76 (ddd, 1H, *J* = 8.7, 7.3 and 1.6 Hz), 7.51(d, 1H, *J* = 8.4 Hz), 7.45 (td, 1H, *J* = 7.5 and 1.1 Hz), 7.43-7.37 (m, 2H), 7.20-7.13 (m, 2H), 4.53 (d, 2H, *J* = 6.1 Hz); $^{13}C\{^1H\}$-NMR (150 MHz, DMSO-d_6) δ 162.5, 161.8, 160.9, 160.8, 154.4, 148.0, 135.7, 135.6, 134.6, 130.7, 130.0, 129.9, 125.6, 119.5, 118.9, 116.6, 115.6, 115.5, 42.5.

***N*-(2-methoxybenzyl)-2-oxo-*2H*-chromene-3-carboxamide (3j).** As white crystals; yield-85%; Mp 214-216 °C, [Lit: Mp 208-210 °C] [24]; FT-IR ν_{max}/cm^{-1} 3346, 3057, 1702, 1651, 1607, 1527, 1107, 738; ^1H NMR (600 MHz, $CDCl_3$) δ 9.20 (t, 1H, *J* = 5.7 Hz, NH), 8.92 (s, 1H), 7.38-7.24 (m, 7H), 7.19 (dd, 1H, *J* = 8.0 and 1.5 Hz), 4.66 (d, 2H, *J* = 5.9 Hz), 3.99 (s,

3H); $^{13}C\{^1H\}$-NMR (150 MHz, $CDCl_3$) δ 161.6, 160.9, 148.8, 147.1, 144.1, 137.9, 128.7, 127.7, 127.5, 125.2, 120.9, 119.3, 118.6, 115.6, 56.4, 43.9.

***N*-(2-(1*H*-indol-3-yl)ethyl)-2-oxo-2*H*-chromene-3-carboxamide (3k).** As brown solid; yield-66%; Mp 185-187 °C, [Lit : Mp 184-188 °C] [44]; FT-IR ν_{max}/cm^{-1} 3311, 1732, 1713, 1644, 1604, 1567, 1534, 735; ^1H NMR (600 MHz, DMSO-d_6) δ 10.85 (s, 1H, NH), 8.88 (s, 1H), 8.83 (t, 1H, J = 5.7 Hz, NH), 7.98 (dd, 1H, J = 7.8 and 1.6 Hz), 7.75 (ddd, 1H, J = 8.6, 7.3 and 1.6 Hz), 7.62 (dd, 1H, J = 7.9 and 1.1 Hz), 7.50 (d, 1H, J = 8.4 Hz), 7.44 (td, 1H, J = 7.5 and 1.0 Hz), 7.35 (dt, 1H, J = 8.1 and 1.0 Hz), 7.22 (d, 1H, J = 2.3 Hz), 7.08 (ddd, 1H, J = 8.1, 6.9, and 1.2 Hz), 6.99 (ddd, 1H, J = 8.0, 6.9, and 1.0 Hz), 3.64 (td, 2H, J = 7.3 and 5.7 Hz), 2.97 (t, 2H, J = 7.2 Hz); $^{13}C\{^1H\}$-NMR (150 MHz, DMSO-d_6) δ 161.5, 160.8, 154.3, 147.9, 136.8, 134.5, 130.7, 127.6, 125.6, 123.3, 121.5, 119.4, 118.9, 118.8, 118.7, 116.6, 111.9, 111.9, 40.4, 25.4.

2-oxo-*N*-phenyl-2*H*-chromene-3-carboxamide (3l). As green solid; yield-70%; Mp 256-257 °C, [Lit: Mp 255.4-256.6 °C] [45]; FT-IR ν_{max}/cm^{-1} 3251, 1713, 1699, 1593, 1549, 1442, 1202, 743; ^1H NMR (600 MHz, $CDCl_3$) δ 10.77 (s, 1H, NH), 8.96 (s, 1H), 7.67 (d, 3H, J = 8.2 Hz), 7.63 (ddd, 1H, J = 8.6, 7.3 and 1.6 Hz), 7.38 (d, 1H, J = 8.4 Hz), 7.35 (d, 1H, J = 7.6 Hz), 7.34-7.30 (m, 2H), 7.10 (td, 1H, J = 7.4 and 1.1 Hz); $^{13}C\{^1H\}$-NMR (150 MHz, $CDCl_3$) δ 161.9, 159.3, 154.5, 149.0, 137.7, 134.4, 130.0, 129.1, 125.5, 124.9, 120.6, 118.7, 118.7, 116.8.

(*S*)-2-oxo-*N*-(1-phenylethyl)-2*H*-chromene-3-carboxamide (3m). As white solid; yield-50%; Mp 135-137 °C; FT-IR ν_{max}/cm^{-1} 3310, 1707, 1655, 1611, 1567, 1520, 760, 697, 536; $[\alpha]_D^{20}$= +7.5 (c 1.0, $CHCl_3$); ^1H NMR (600 MHz, DMSO-d_6) δ 9.02 (d, 1H, J = 7.8 Hz, NH),

8.82 (s, 1H), 7.97 (dd, 1H, J = 7.8 and 1.6 Hz), 7.75 (ddd, 1H, J = 8.7, 7.3 and 1.6 Hz), 7.52 (d, 1H, J = 8.4 Hz), 7.44 (td, 1H, J = 7.5 and 1.0 Hz), 7.44-7.39 (m, 2H), 7.36 (dd, 2H, J = 8.5 and 6.9 Hz), 7.30-7.24 (m, 1H), 5.15 (p, 1H, J = 7.1 Hz); 1.51 (d, 3H, J = 7.0 Hz); $^{13}C\{^1H\}$-NMR (150 MHz, DMSO-d_6) δ 161.0, 160.9, 154.3, 147.8, 144.2, 134.6, 130.7, 129.0, 127.5, 126.5, 125.6, 119.7, 118.9, 116.6, 49.2, 22.9.

***N*-isopropyl-2-oxo-2*H*-chromene-3-carboxamide (3n).** As white solid; yield-43%; Mp 133-135 °C, [Lit: Mp 113-117 °C] [24]; FT-IR ν_{max}/cm^{-1} 3302, 2970, 1702, 1655, 1609, 1565, 1525, 796, 760, 637; ^1H NMR (600 MHz, in $CDCl_3$) δ 8.91 (s, 1H), 8.68 (br s, 1H, NH), 7.69 (dd, 1H, J = 7.7 and 1.6 Hz), 7.66 (ddd, 1H, J = 8.7, 7.4 and 1.6 Hz), 7.43-7.34 (m, 2H), 4.26 (dq, 1H, J = 13.2, 6.6 Hz), 1.29 (d, 6H, J = 6.6 Hz); $^{13}C\{^1H\}$-NMR (150 MHz, in $CDCl_3$) δ 161.4, 160.5, 154.4, 148.1, 133.9, 129.7, 125.2, 118.7, 116.6, 41.9, 22.6.

***N,N*-dimethyl-2-oxo-2*H*-chromene-3-carboxamide (7a).** As pale yellow solid; yield-23%; Mp 148-149 °C, [Lit: Mp 150-152 °C] [24]; FT-IR ν_{max}/cm^{-1} 3064, 1711, 1637, 1605, 1569, 1196, 1012, 761; ^1H NMR (600 MHz, $CDCl_3$) δ 7.85 (s, 1H), 7.52 (ddd, 1H, J = 8.6, 7.3 and 1.6 Hz), 7.47 (dd, 1H, J = 7.8 and 1.6 Hz), 7.29 (d, 1H, J = 8.4 Hz), 7.26 (td, 1H, J = 7.5 and 1.1 Hz), 3.05 (s, 3H), 2.95 (s, 3H); $^{13}C\{^1H\}$-NMR (150 MHz, $CDCl_3$) δ 165.0, 157.9, 154.1, 142.9, 132.8, 128.5, 125.8, 124.9, 118.3, 116.8, 38.4, 35.3.

8-methoxy-*N,N*-dimethyl-2-oxo-2*H*-chromene-3-carboxamide (7b). As white solid; yield-49%; Mp 160-162 °C, [Lit: Mp 162-164 °C] [24]; FT-IR ν_{max}/cm^{-1} 3410, 2942, 1710, 1638, 1612, 1579, 1260, 1198, 1109, 771, 734; ^1H NMR (600 MHz, $CDCl_3$) δ 7.90 (s, 1H), 7.25 (t, 1H, J = 8.0 Hz), 7.12 (ddd, 2H, J = 15.6, 8.0 and 1.3 Hz), 3.98 (s, 3H), 3.11 (s, 3H),

3.01 (s, 3H); $^{13}C\{^1H\}$-NMR (150 MHz, $CDCl_3$) δ 165.0, 157.3, 147.1, 143.8, 143.2, 126.0, 124.8, 119.8, 119.0, 114.5, 56.3, 38.4, 35.3.

3-(pyrrolidine-1-carbonyl)-2*H*-chromene-2-one (7c). As yellow solid; yield-22%; Mp 176-178 °C, [Lit : Mp 140-142 °C] [24]; FT-IR ν_{max}/cm^{-1} 3053, 2977, 1738, 1599, 1569, 1454, 1266, 750; ^1H NMR (600 MHz, $CDCl_3$) δ 7.90 (s, 1H), 7.52 (ddd, 1H, *J* = 8.7, 7.3 and 1.6 Hz), 7.48 (dd, 1H, *J* = 7.7 and 1.1 Hz), 7.29 (d, 1H, *J* = 8.3 Hz), 7.25 (td, 1H, *J* = 7.5 and 1.0 Hz), 3.57 (t, 2H, *J* = 6.9 Hz), 3.39 (t, 2H, *J* = 6.6 Hz), 2.00-1.81(m, 4H); DEPTQ$\{^1H\}$-NMR (150 MHz, $CDCl_3$) δ 163.3, 157.8, 154.1, 143.1, 132.8, 128.7, 126.2, 124.9, 118.3, 116.8, 47.6, 46.3, 26.0, 24.3.

3-(piperidine-1-carbonyl)-2*H*-chromene-2-one (7d). As brown solid; yield-40%; Mp 184-186 °C, [Lit[24]: Mp 156-158 °C] [24]; FT-IR ν_{max}/cm^{-1} 3046, 2970, 1711, 1608, 1569, 1439, 1251, 757; ^1H NMR (600 MHz, $CDCl_3$) δ 7.80 (s, 1H), 7.51 (ddd, 1H, *J* = 8.6, 7.3 and 1.6 Hz), 7.46 (dd, 1H, *J* = 7.7 and 1.6 Hz), 7.28 (d, 1H, *J* = 8.3 Hz), 7.25 (td, 1H, *J* = 7.5 and 1.1 Hz), 3.67-3.62 (m, 2H), 3.27 (t, 2H, *J* = 5.5 Hz), 1.54-1.60 (m, 6H); $^{13}C\{^1H\}$-NMR (150 MHz, $CDCl_3$) δ 163.3, 158.1, 154.0, 142.2, 132.6, 128.4, 125.9, 124.8, 118.4, 116.8, 48.4, 43.0, 26.2, 25.4, 24.4.

6-bromo-3-(piperidine-1-carbonyl)-2*H*-chromen-2-one (7e). As white solid; yield-54%; Mp 210-212 °C, [Lit : Mp 219.2-220 °C] [46]; FT-IR ν_{max}/cm^{-1} 3060, 2933, 1730, 1634, 1613, 1562, 1439, 1230, 813; ^1H NMR (600 MHz, $CDCl_3$) δ 7.77 (s, 1H), 7.62-7.67 (br s, 2H), 7.24 (d, 1H, J = 9.3 Hz), 3.70 (br s, 2H), 3.31 (t, 2H, *J* = 5.6 Hz), 1.60-1.65 (m, 6H); $^{13}C\{^1H\}$-NMR (150 MHz, $CDCl_3$) δ 162.7, 157.4, 152.8, 140.7, 135.3, 130.6, 127.1, 119.9, 118.5, 117.4, 48.4, 43.1, 26.2, 25.4, 24.4.

To gain insight into the reaction mechanism, control experiments were conducted. The imine **4a** and the ester **5** were subjected to the reaction condition as mentioned in the text. The ^{1}H and ^{13}C NMR spectral data of these two compounds are given below:

(*E*)-2-(((2-hydroxyethyl)imino)methyl)phenol (4a). Yellow oil; ^{1}H NMR (600 MHz, $CDCl_3$) δ 13.18 (br s, 1H), 8.40 (s, 1H), 7.32-7.27 (m, 2H), 6.96 (d, 1H, *J* = 8.3 Hz), 6.88 (t, 1H, *J* = 7.5 Hz), 3.93 (t, 1H, *J* = 5.1 Hz), 3.76 (t, 1H, *J* = 5.2 Hz), 1.65 (br s, 2H); ^{13}C{^{1}H}-NMR (150 MHz, $CDCl_3$) δ 167.0, 161.1, 132.5, 131.4, 118.7, 118.7, 117.1, 62.2, 61.8.

ethyl 2-oxo-2*H*-chromene-3-carboxylate (5). white solid; ^{1}H NMR (600 MHz, $CDCl_3$) δ 8.53 (s, 1H), 7.68-7.60 (m, 2H), 7.38-7.31 (m, 2H), 4.42 (q, 2H, *J* = 7.1 Hz), 1.42 (1, 2H, *J* = 7.1 Hz); ^{13}C{^{1}H}-NMR (150 MHz, $CDCl_3$) δ 163.1, 156.7, 155.2, 148.6, 134.3, 129.5, 124.8, 118.4, 117.9, 116.8, 62.0, 14.2.

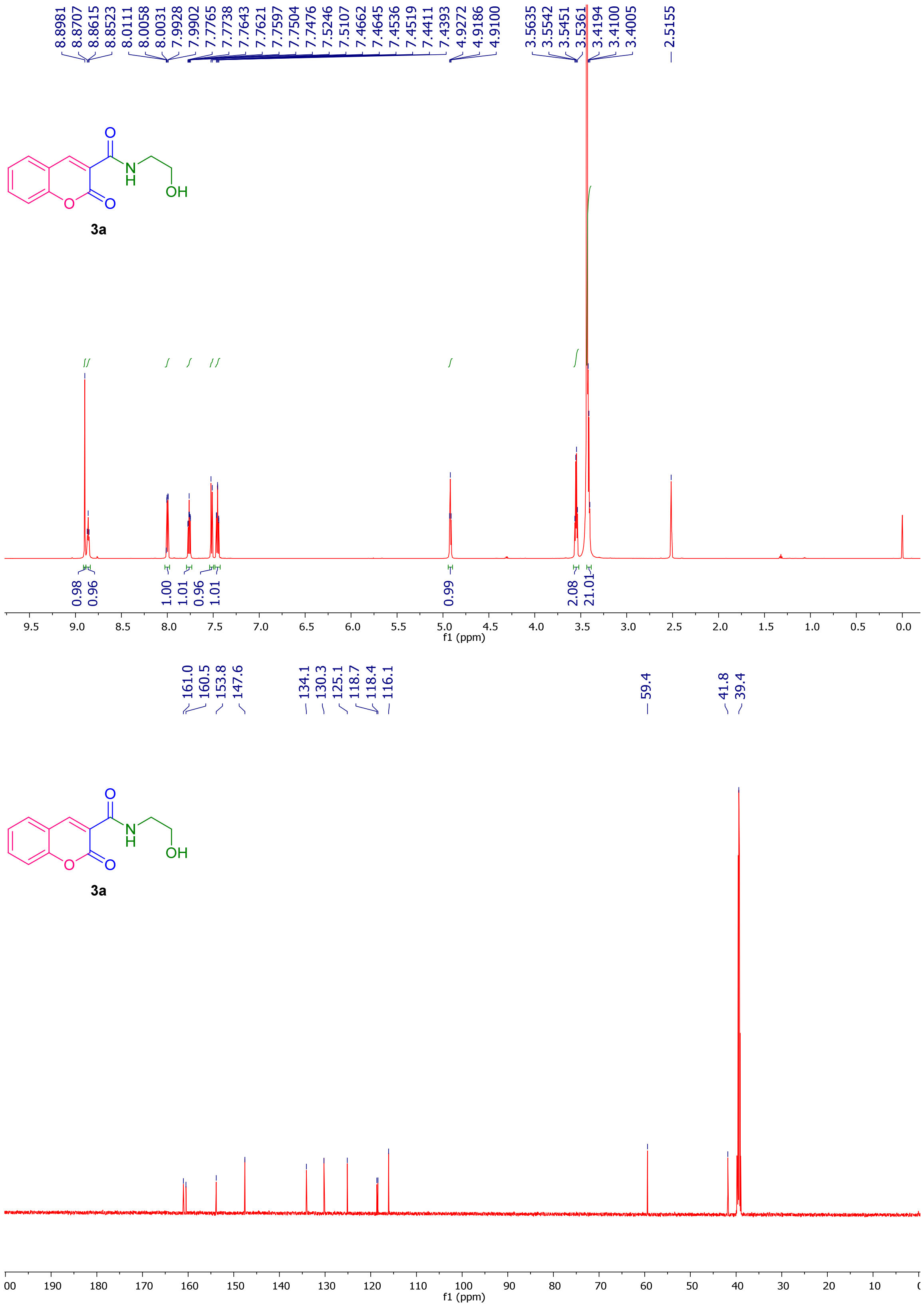

Figure S1. ¹H (600 MHz), ¹³C{¹H} (150 MHz) NMR spectra of **3a** in DMSO-*d*6.

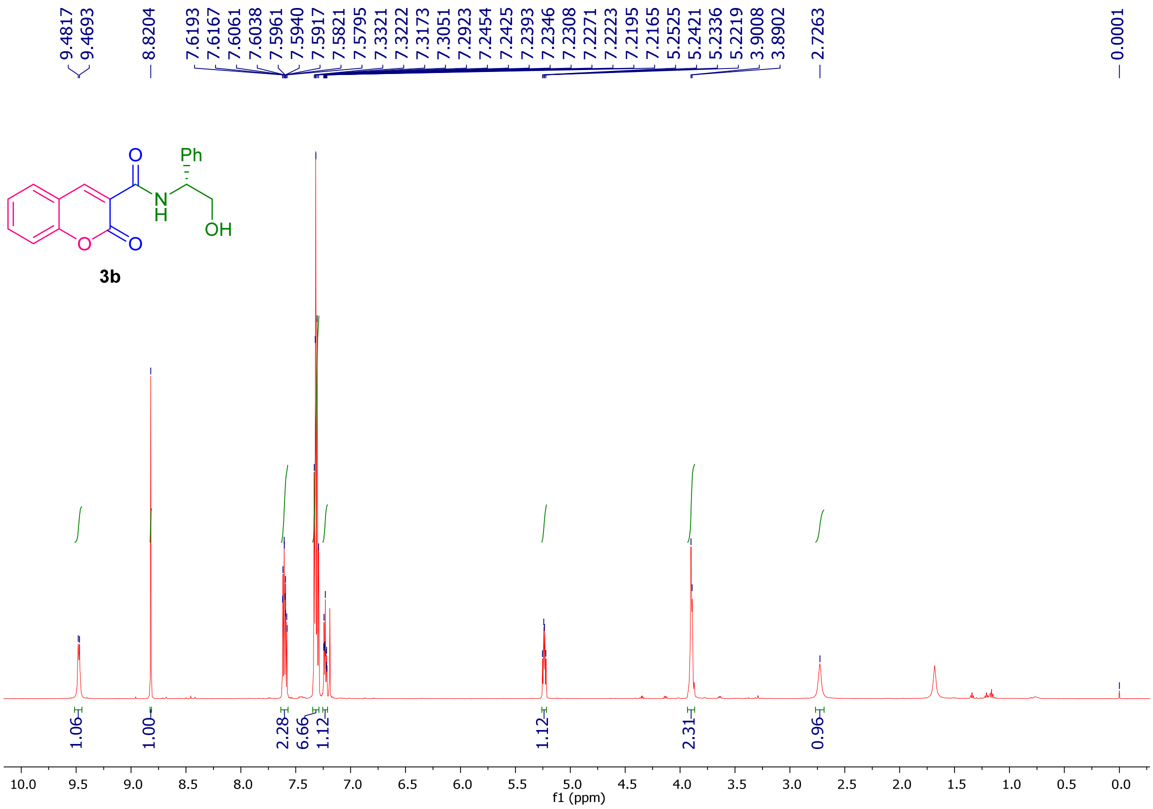

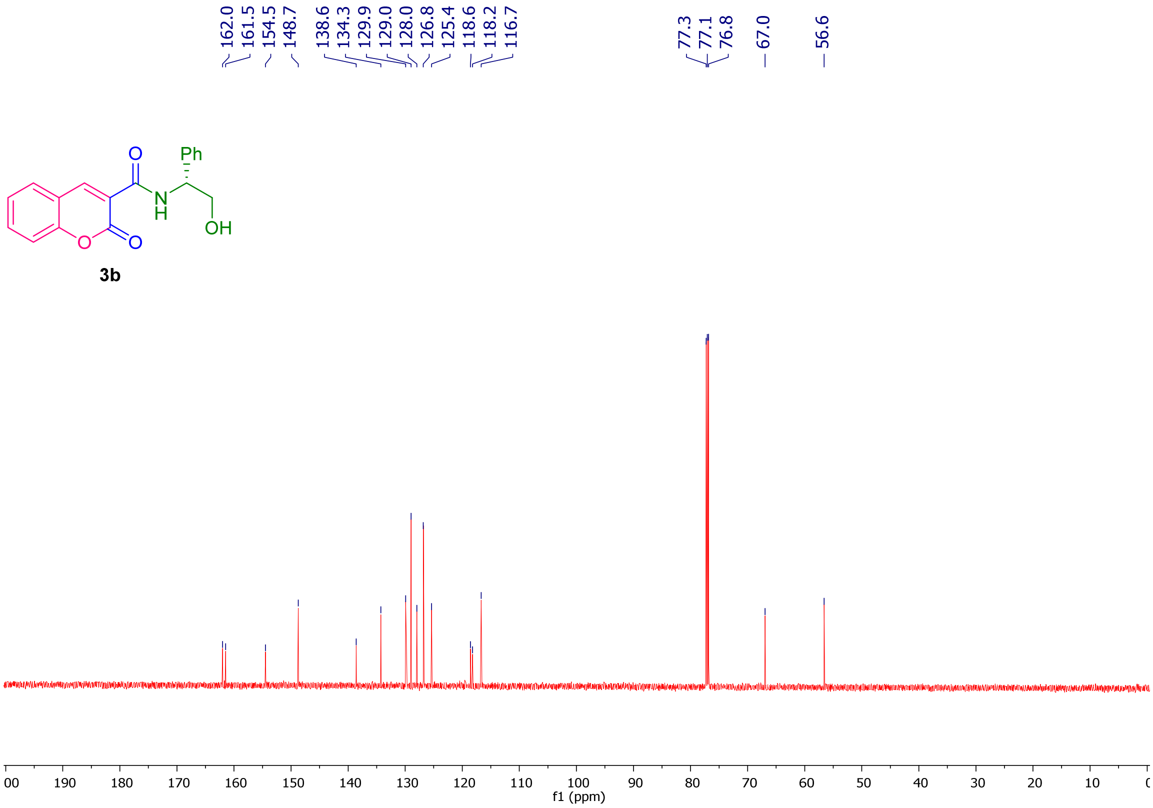

Figure S2. ^{1}H (600 MHz), ^{13}C{^{1}H} (150 MHz) NMR spectra of **3b** in $CDCl_3$.

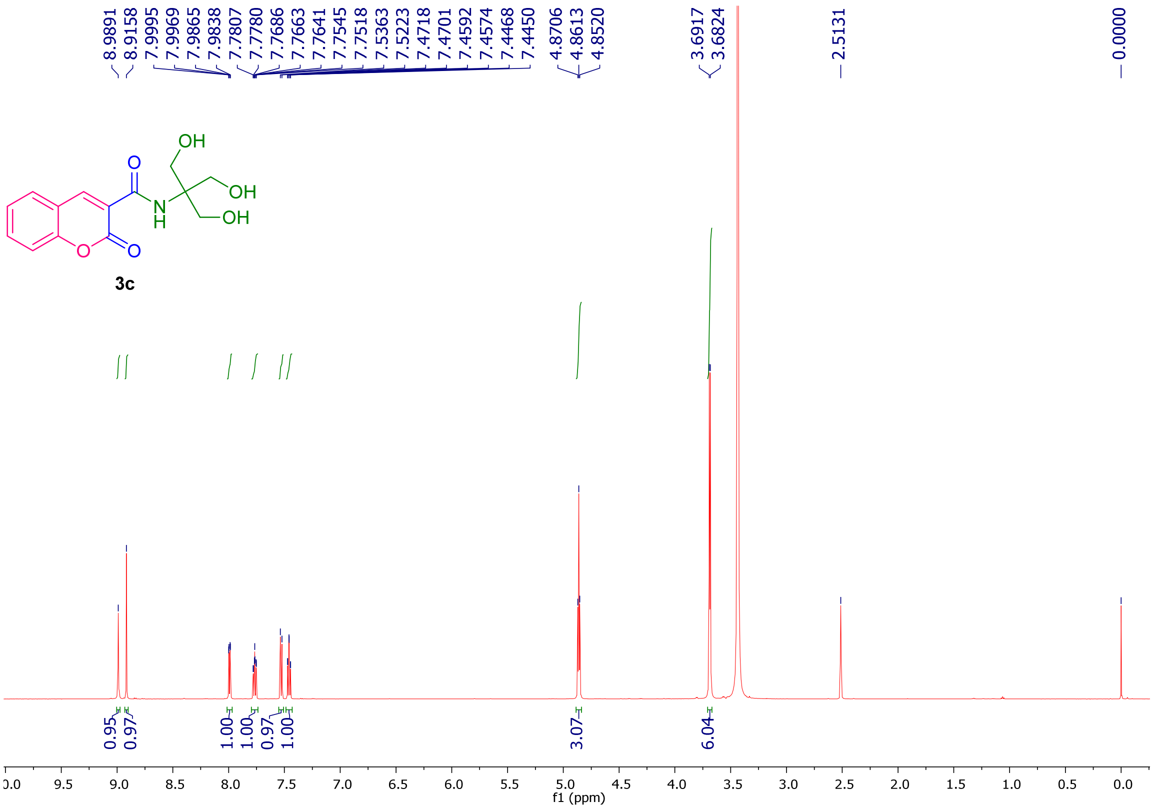

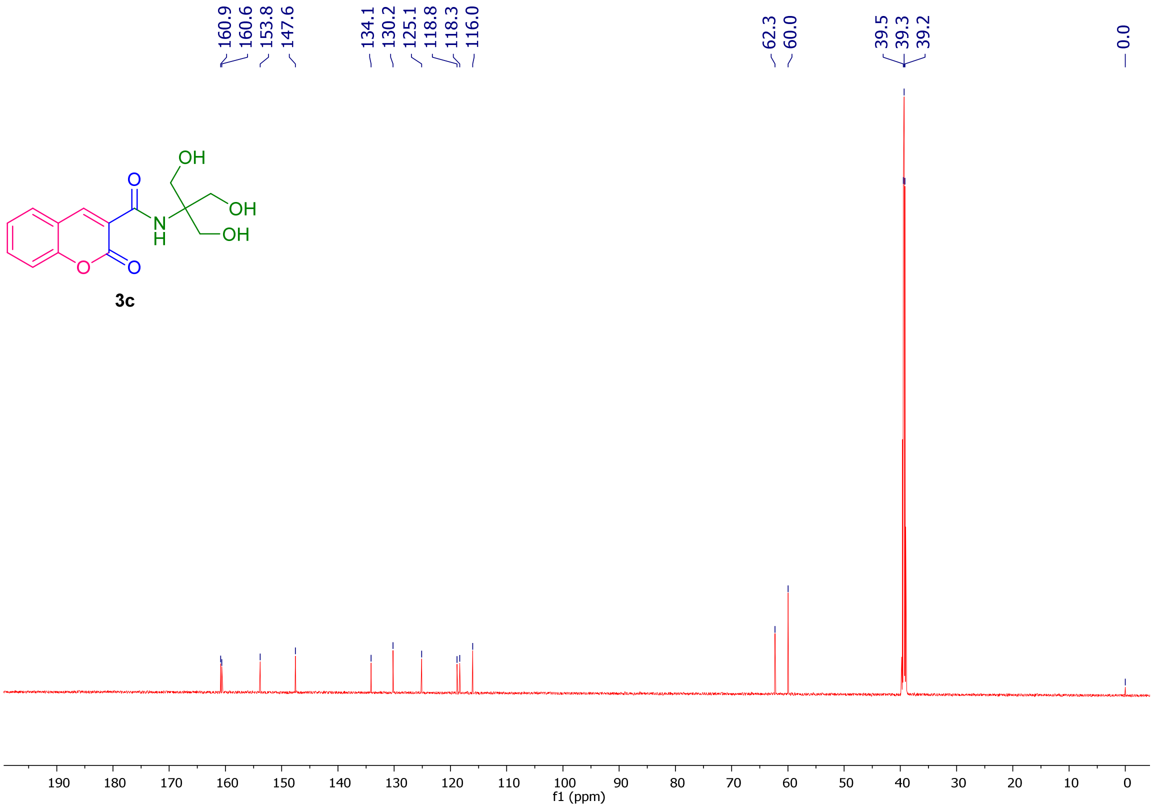

Figure S3. ^{1}H (600 MHz), $^{13}C\{^{1}H\}$ (150 MHz) NMR spectra of **3c** in DMSO-*d*6.

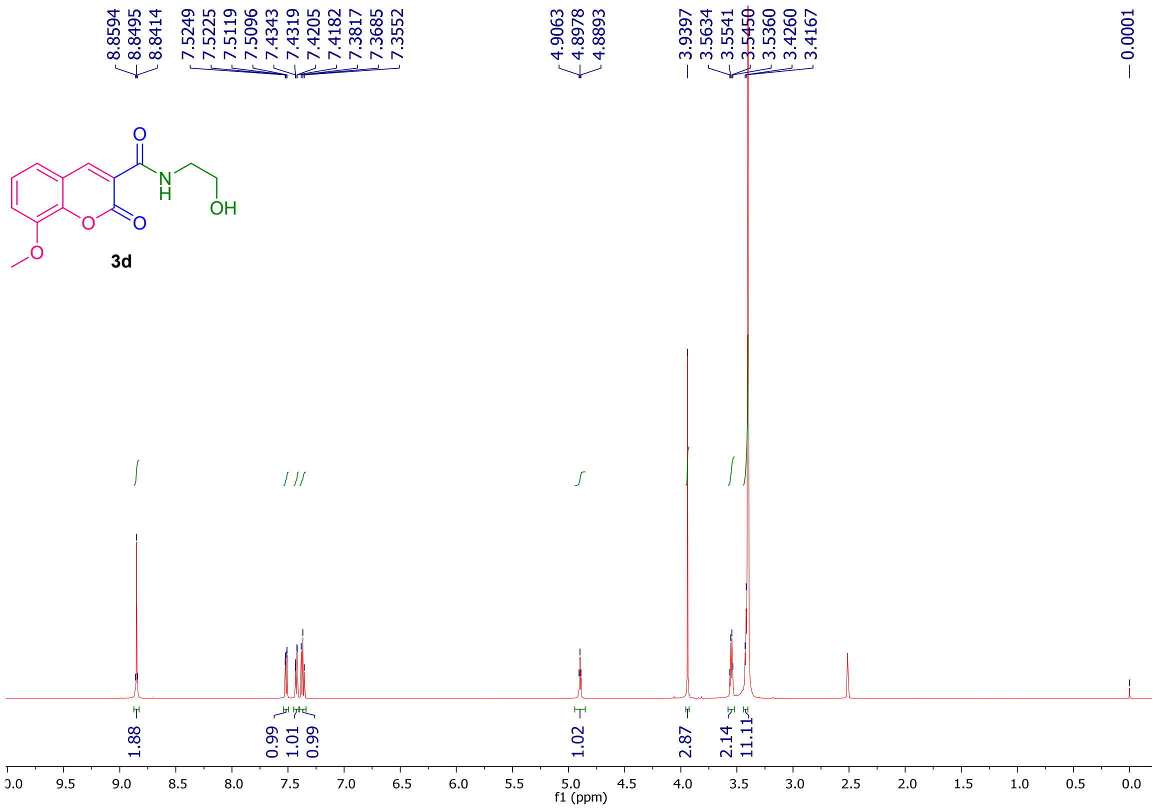

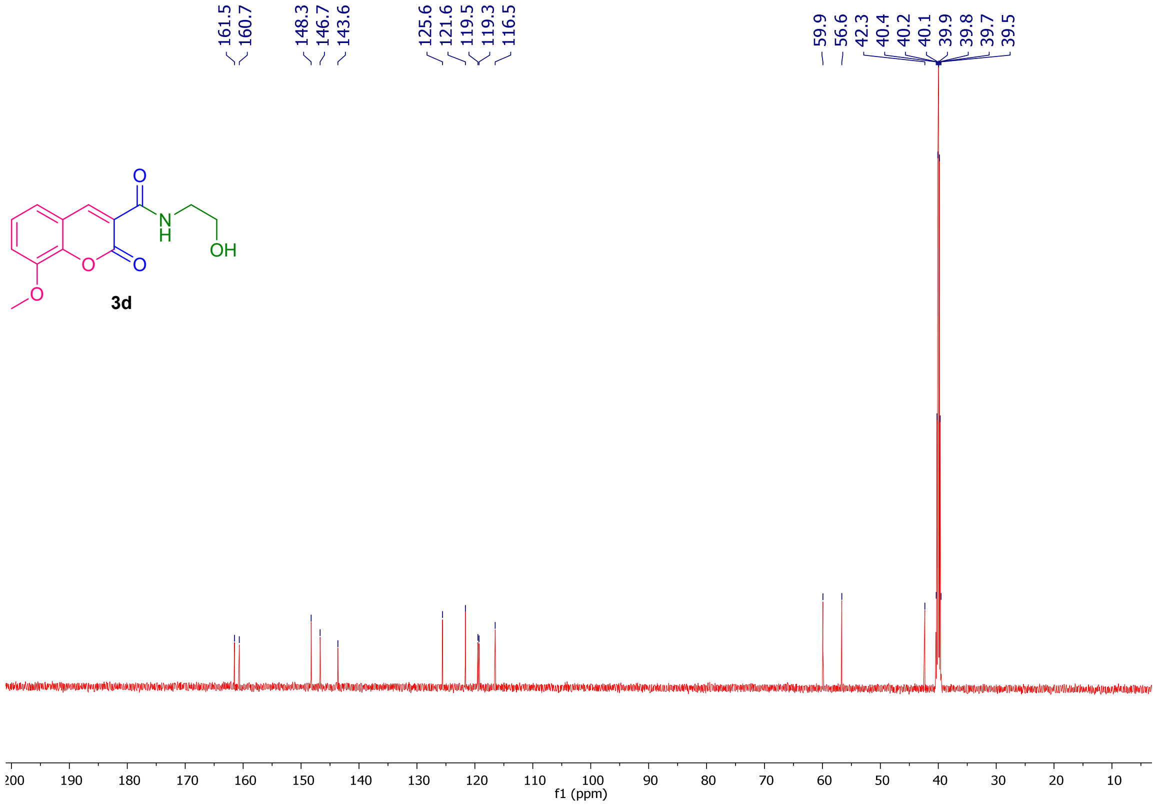

Figure S4. ^{1}H (600 MHz), $^{13}C\{^1H\}$ (150 MHz) NMR spectra of **3d** in DMSO-*d*$_6$.

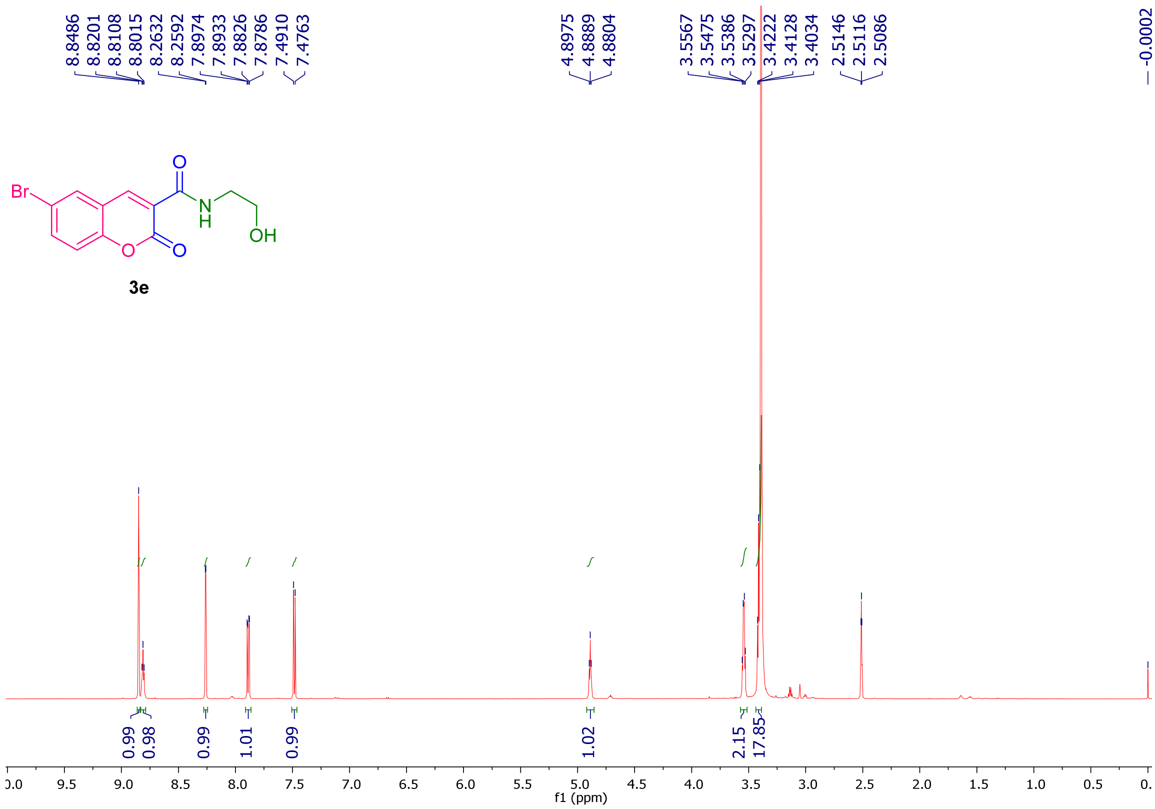

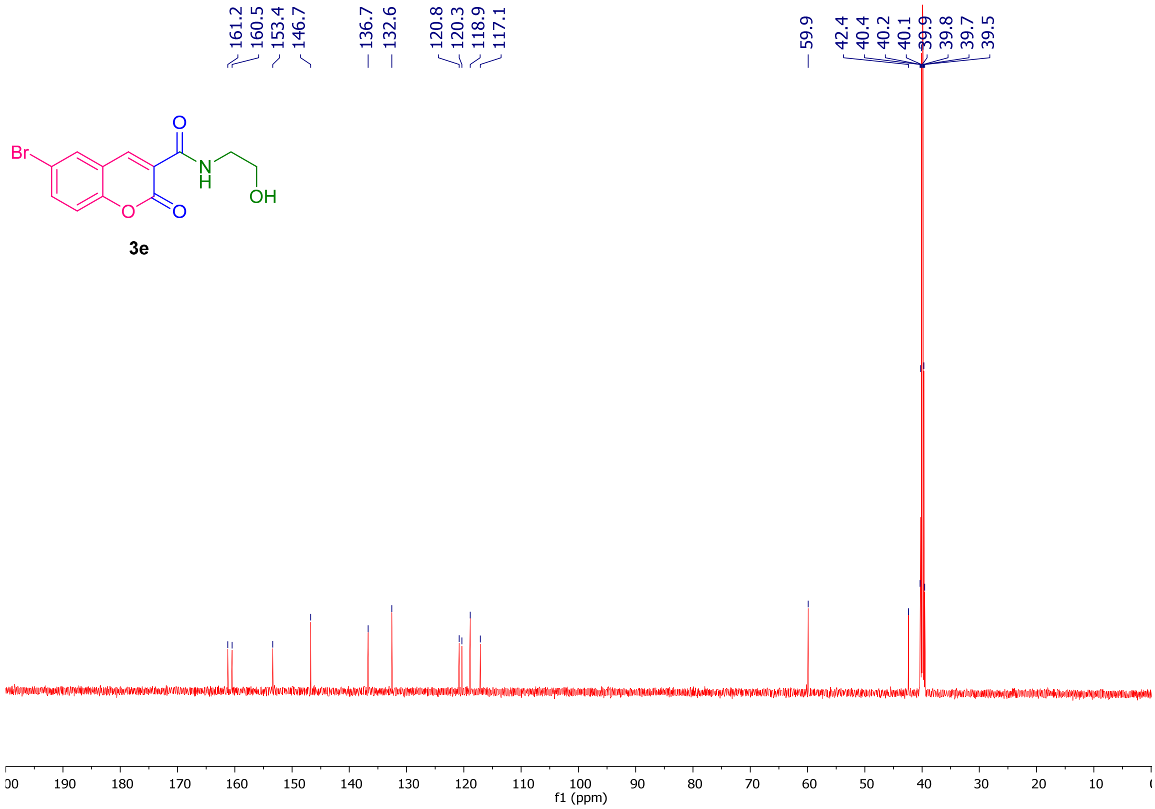

Figure S5. ^{1}H (600 MHz), $^{13}C\{^{1}H\}$ (150 MHz) NMR spectra of **3e** in DMSO-d_6

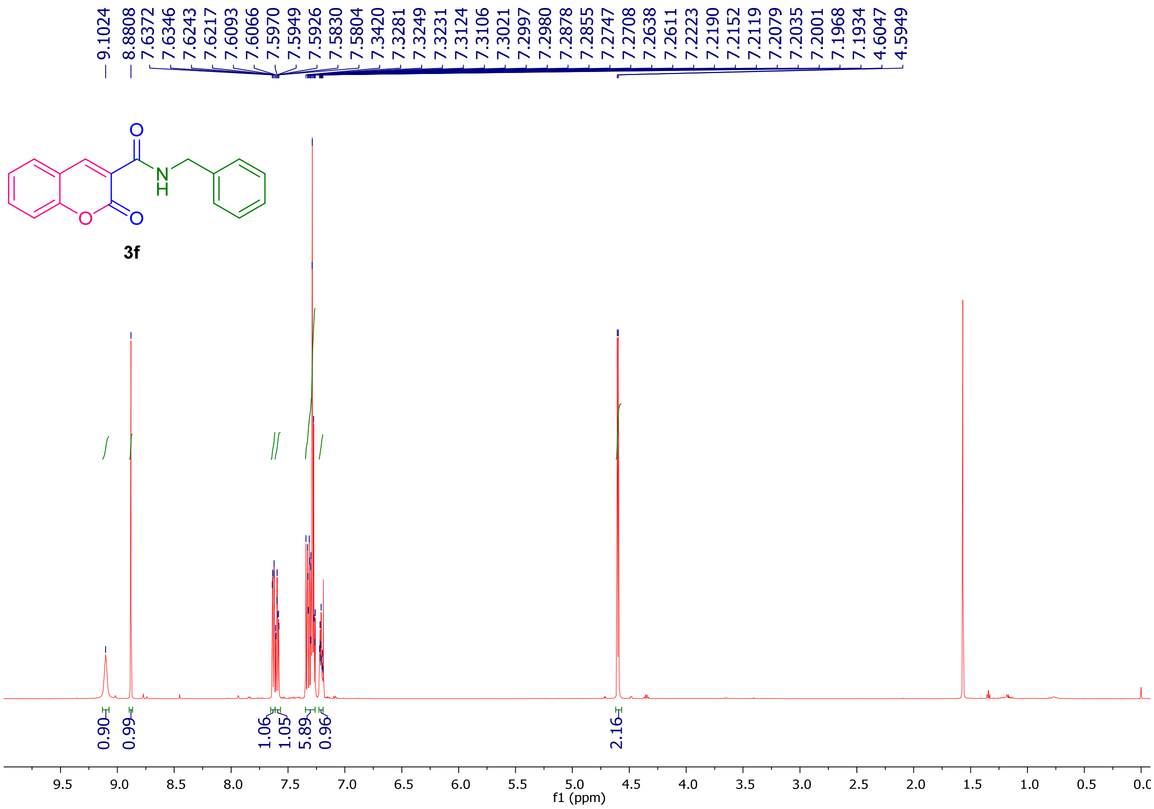

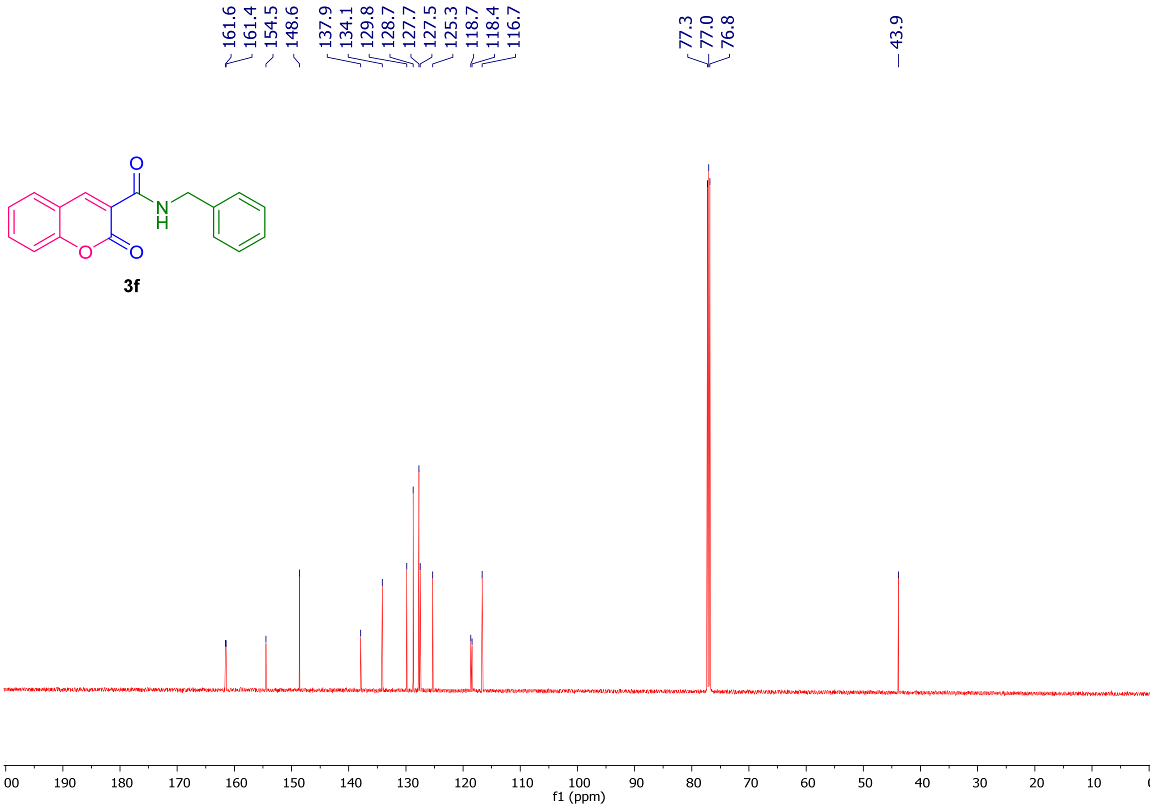

Figure S6. ^{1}H (600 MHz), ^{13}C{^{1}H} (150 MHz) NMR spectra of **3f** in $CDCl_3$

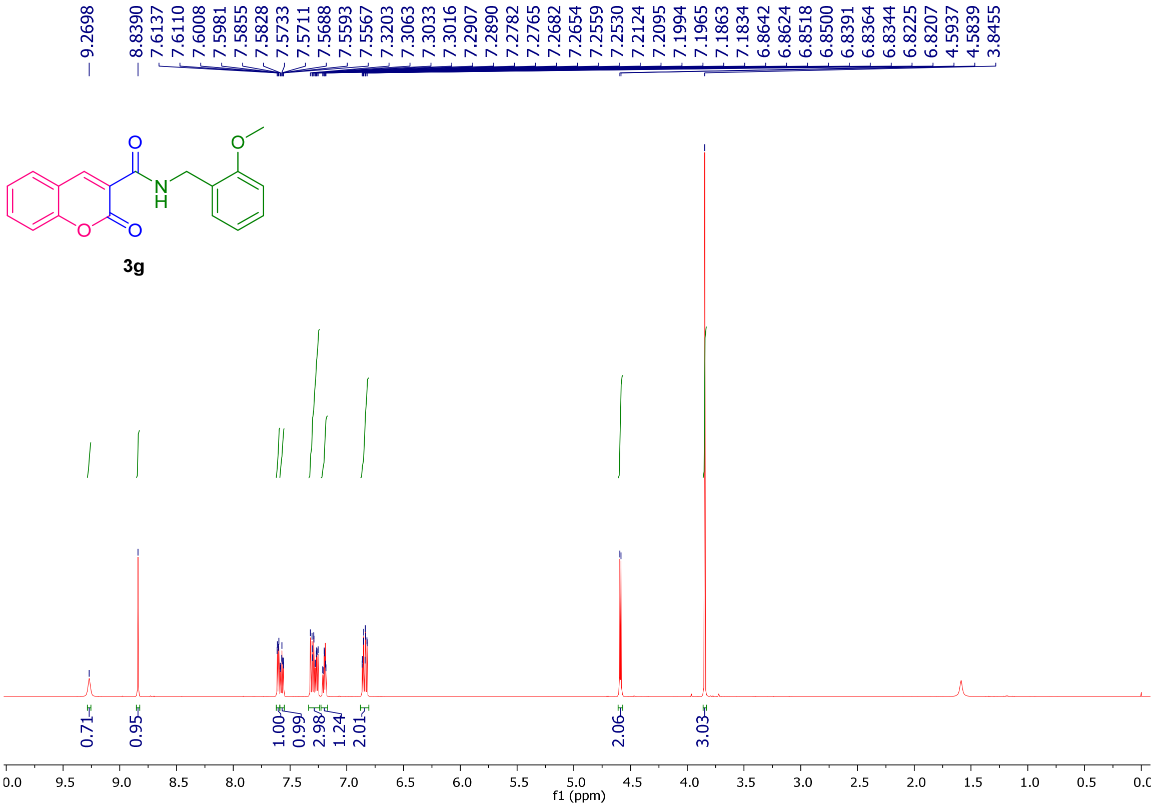

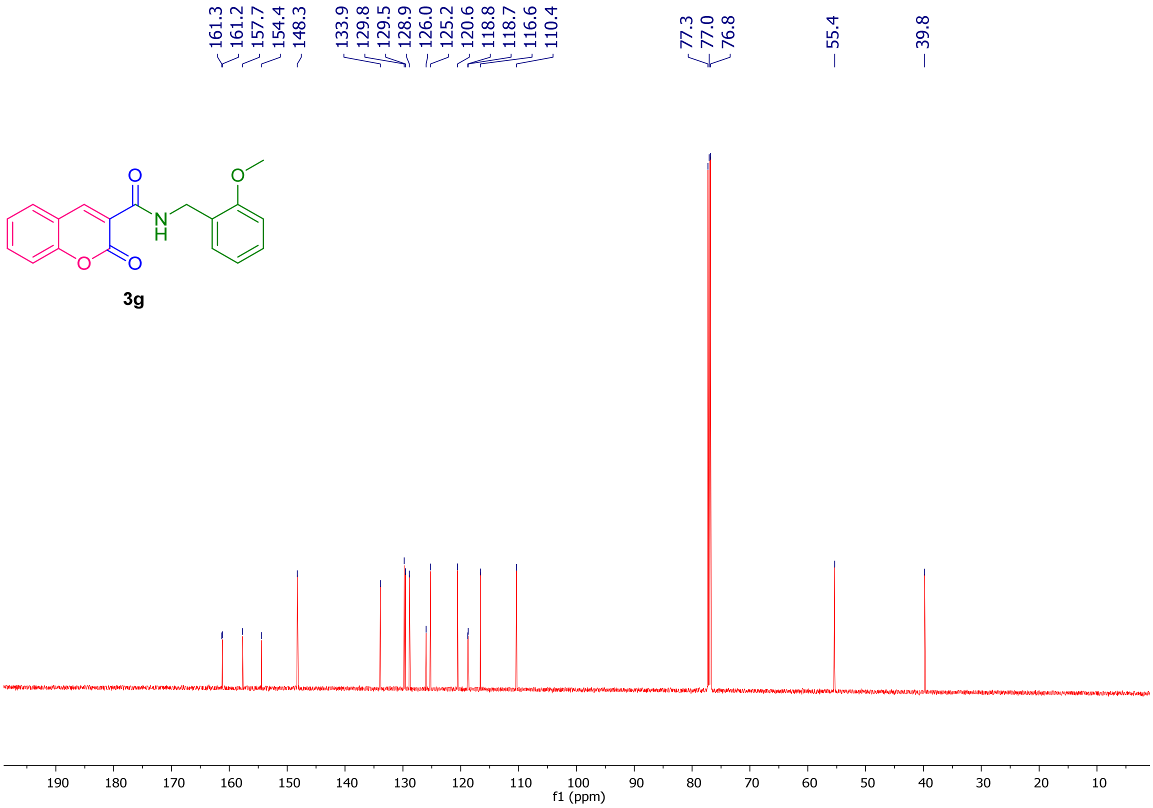

Figure S7. ^{1}H (600 MHz), ^{13}C{^{1}H} (150 MHz) NMR spectra of **3g** in $CDCl_3$.

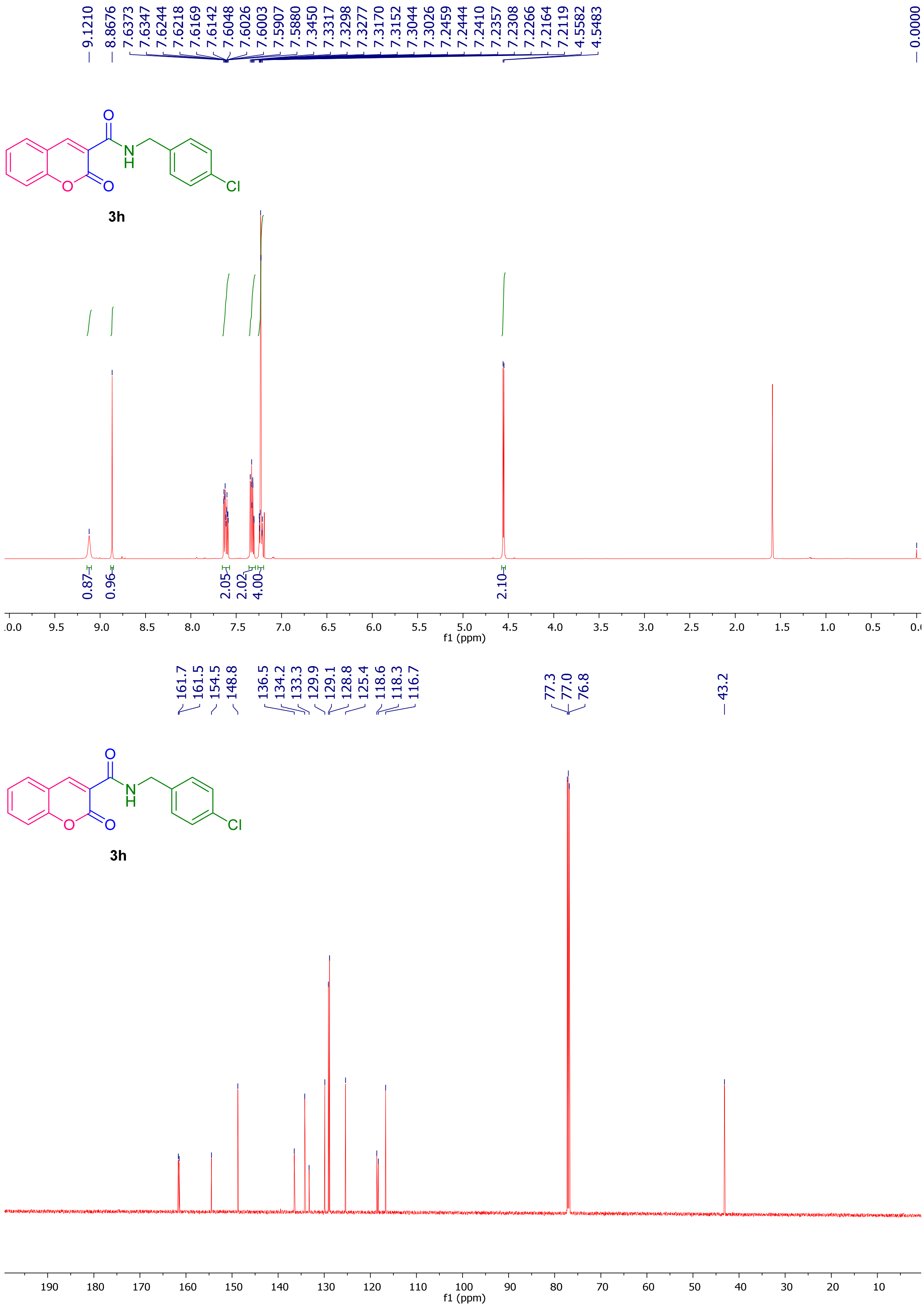

Figure S8. ^{1}H (600 MHz), ^{13}C{^{1}H} (150 MHz) NMR spectra of **3h** in $CDCl_3$.

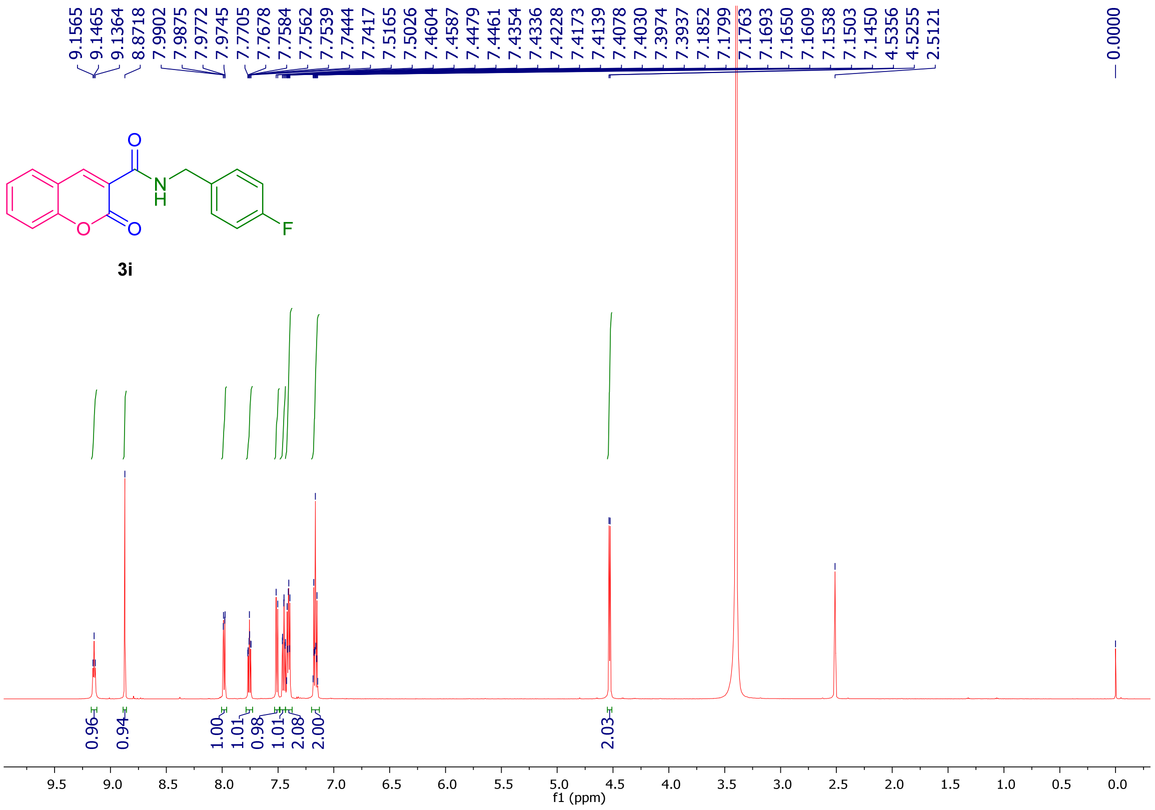

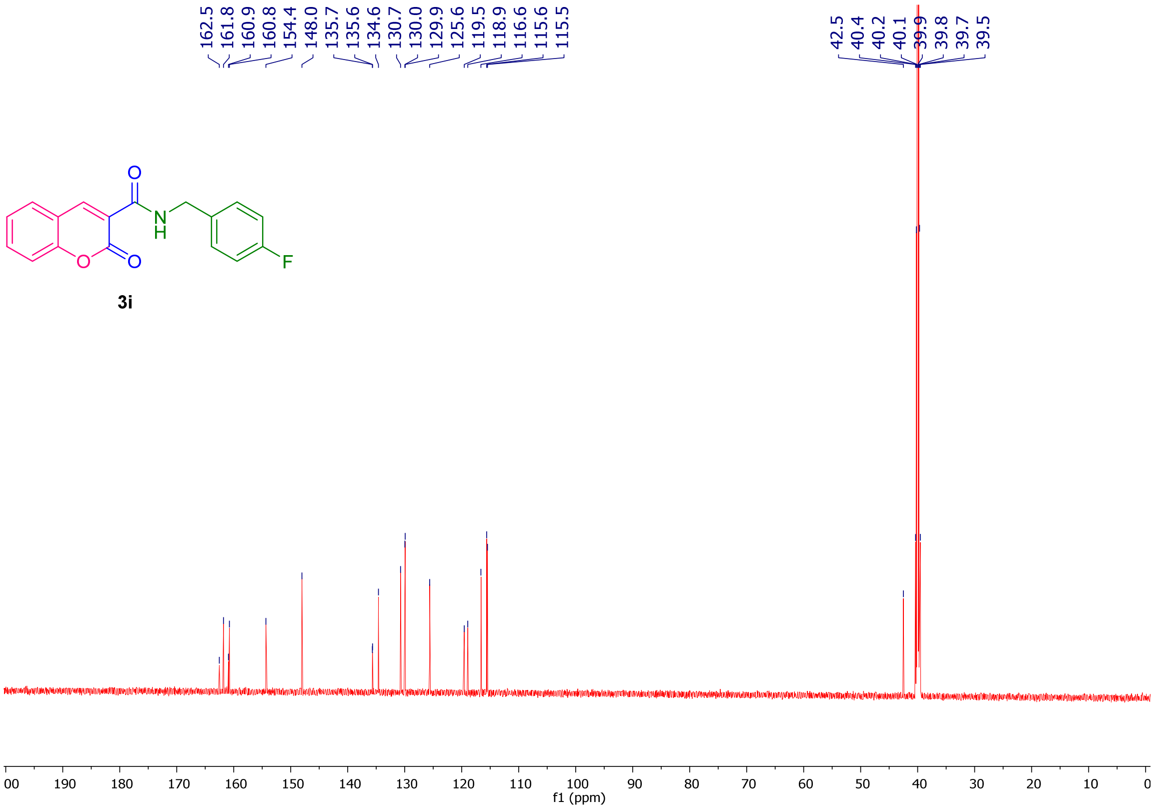

Figure S9. ^{1}H (600 MHz), $^{13}C\{^{1}H\}$ (150 MHz) NMR spectra of **3i** in DMSO-d_6.

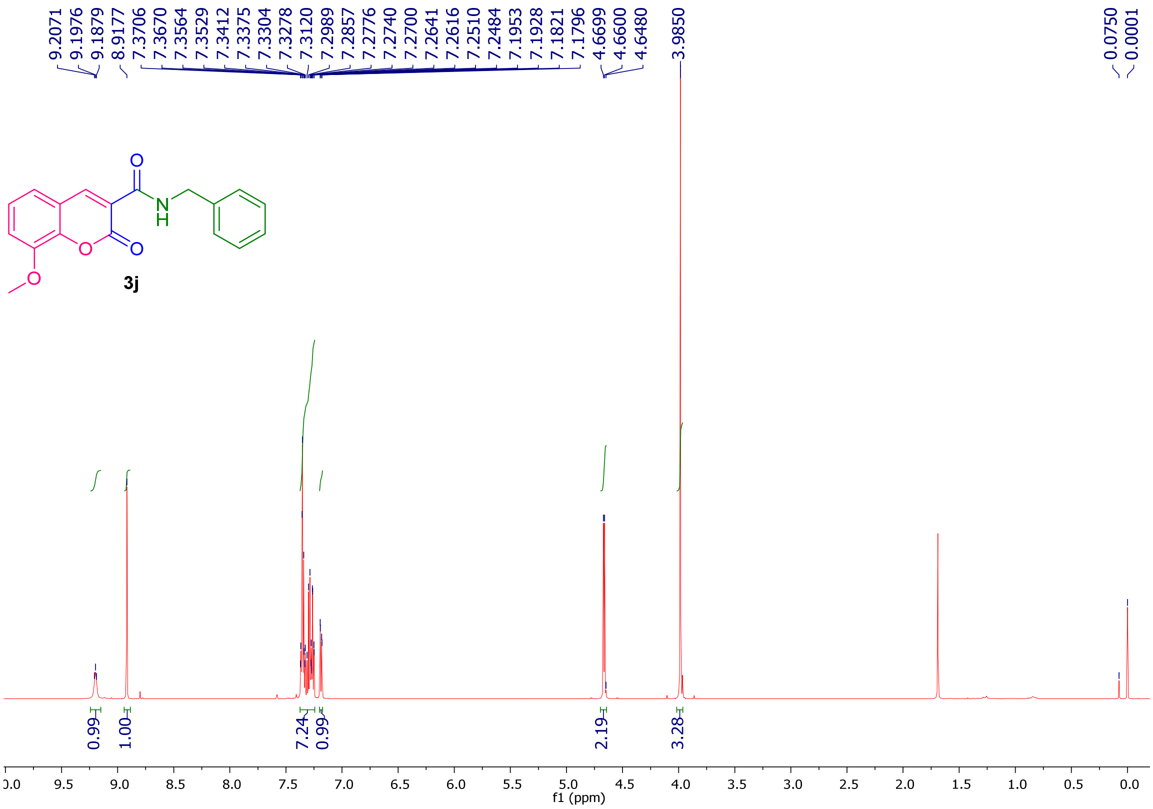

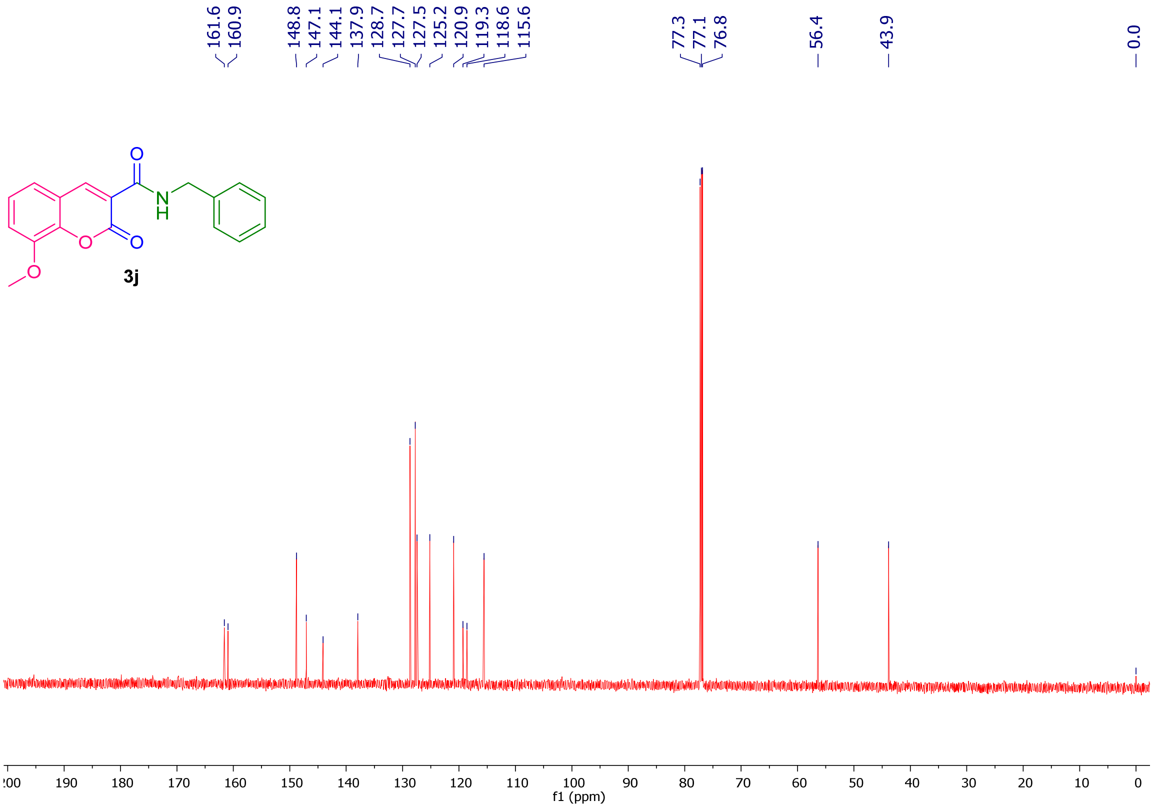

Figure S10. ^{1}H (600 MHz), ^{13}C{^{1}H} (150 MHz) NMR spectra of **3j** in $CDCl_3$

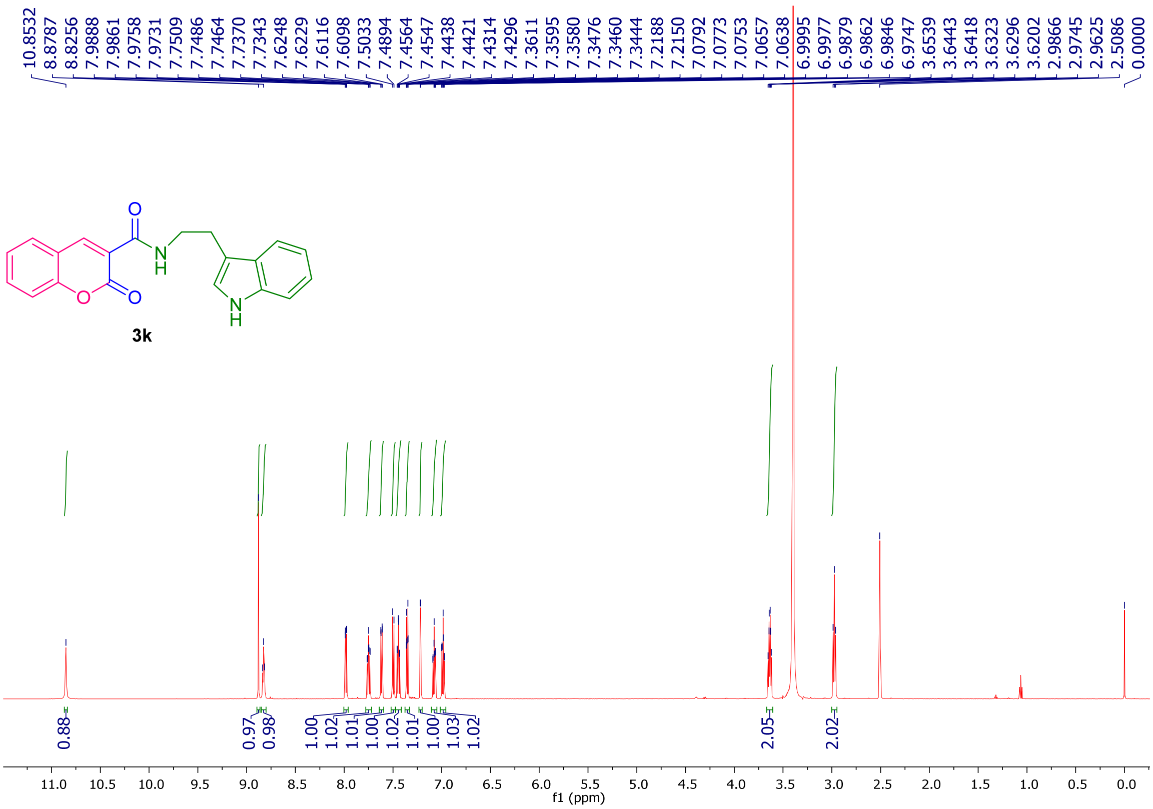

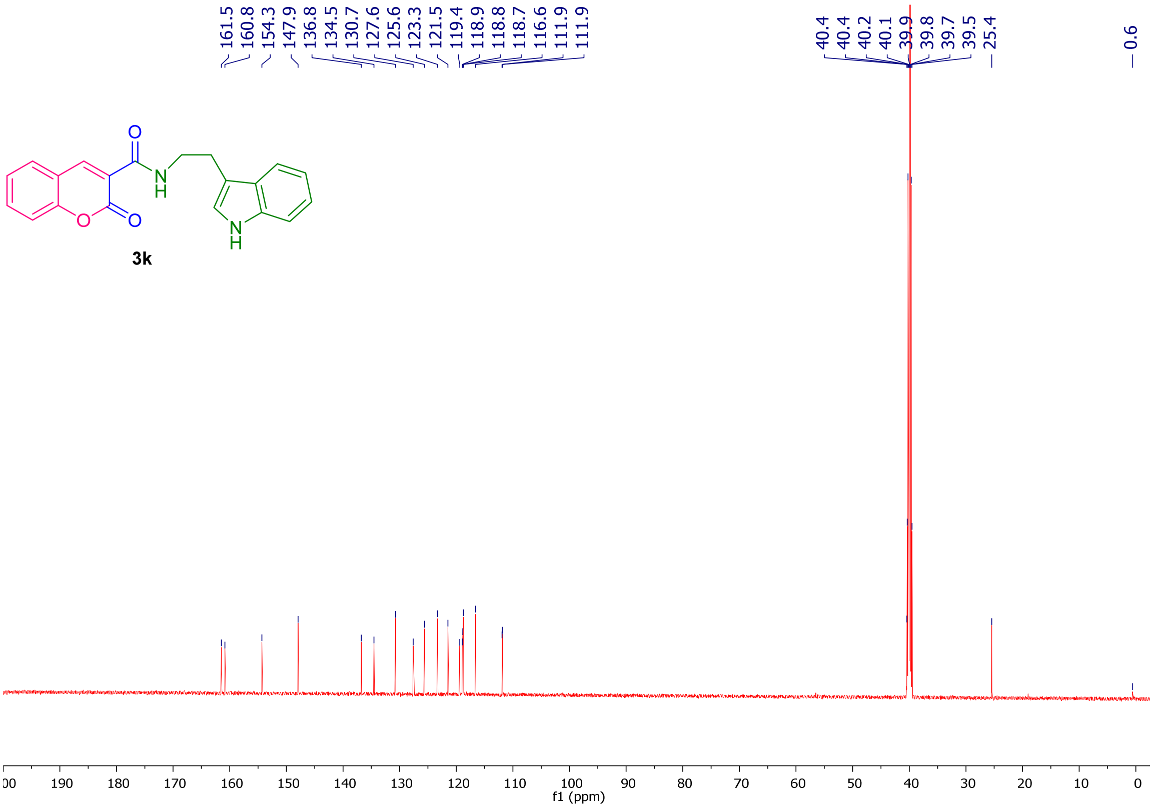

Figure S11. ^{1}H (600 MHz), $^{13}C\{^{1}H\}$ (150 MHz) NMR spectra of **3k** in DMSO-d_6

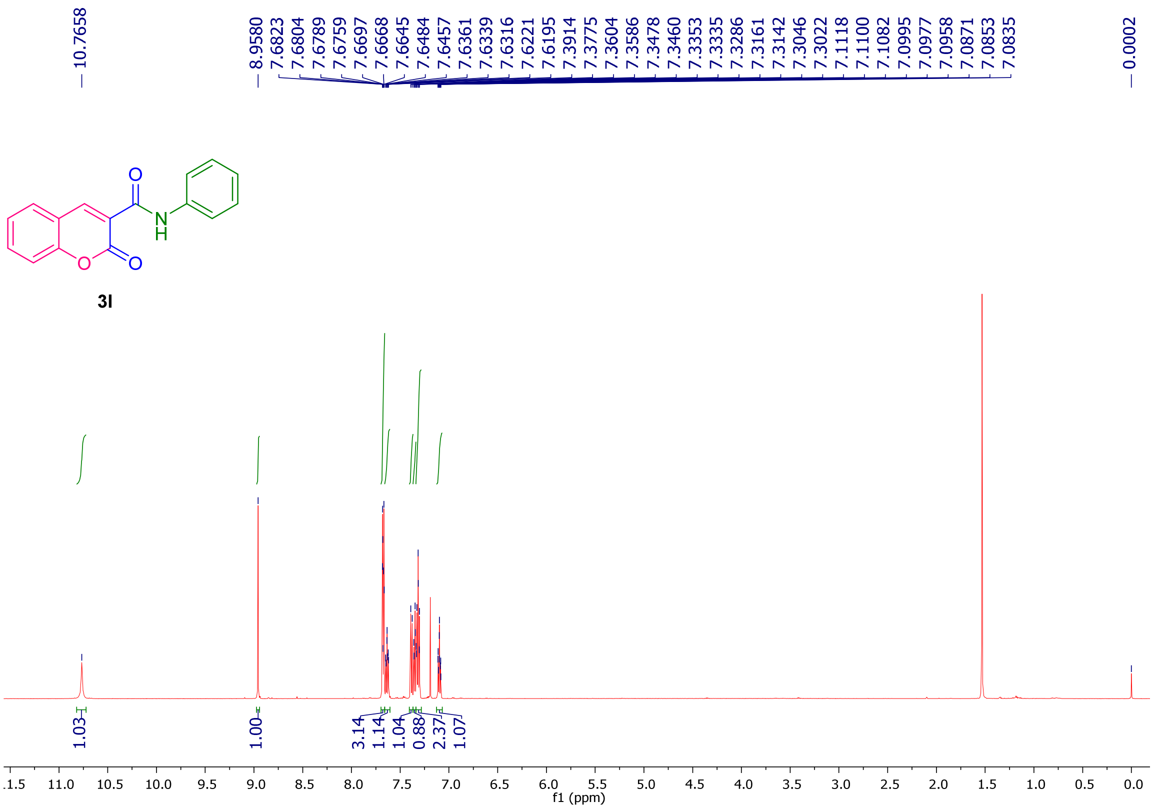

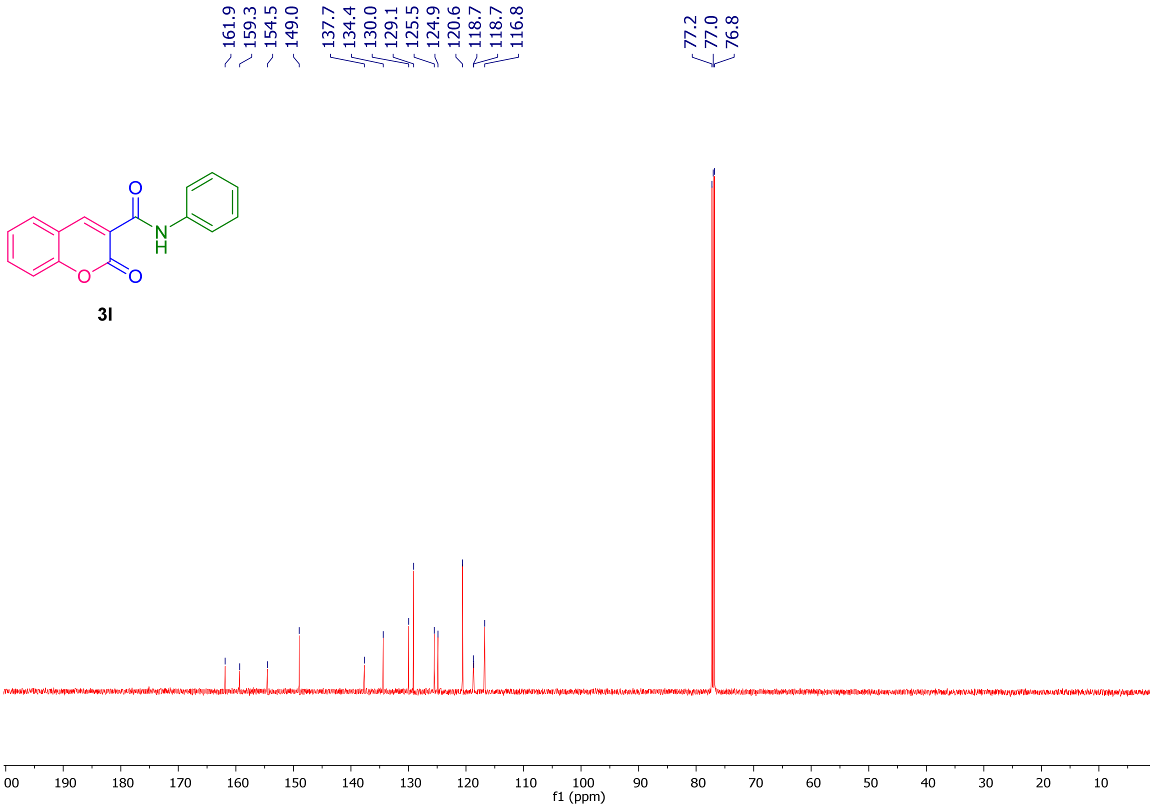

Figure S12. ^{1}H (600 MHz), ^{13}C{^{1}H} (150 MHz) NMR spectra of **3l** in $CDCl_3$.

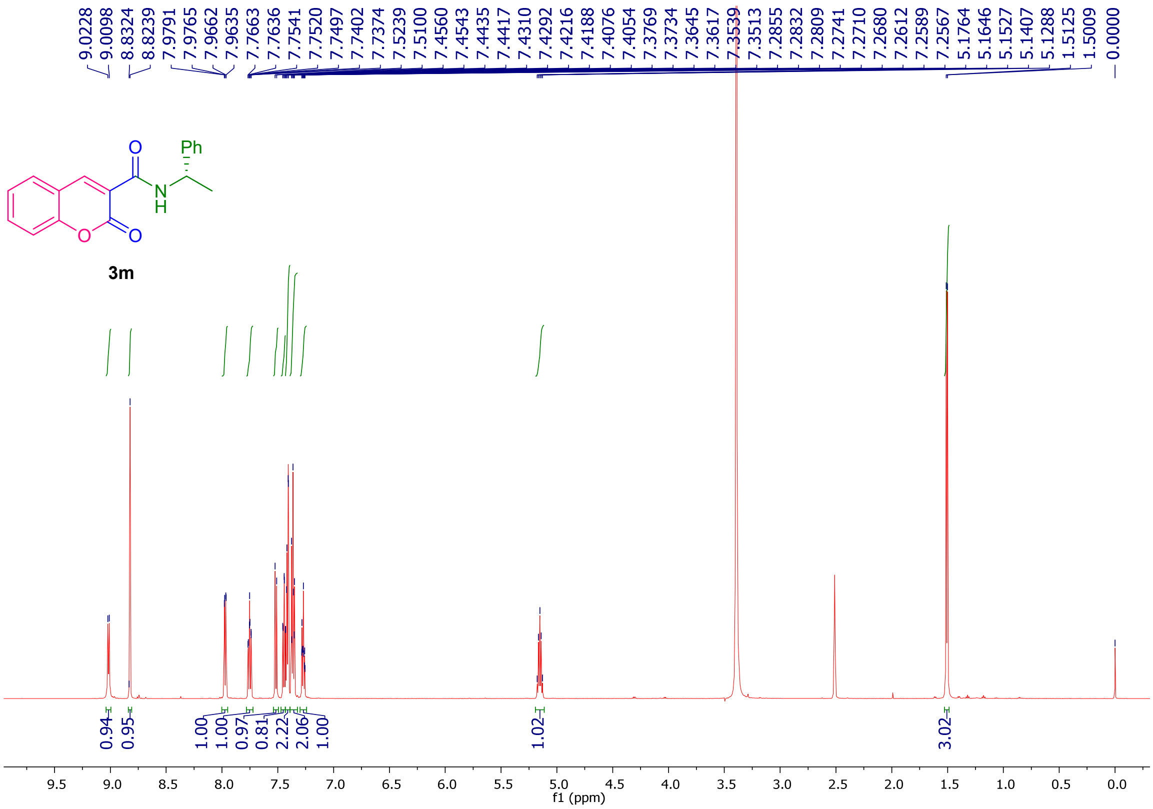

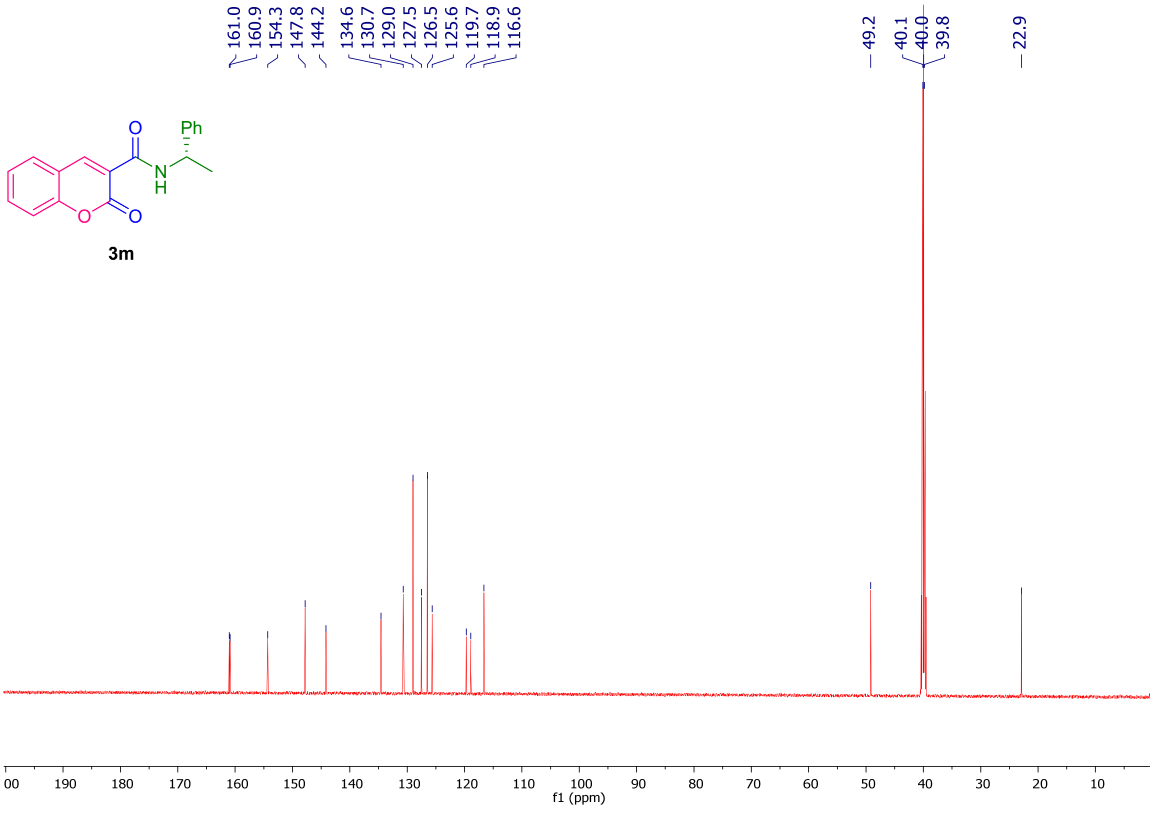

Figure S13. ^{1}H (600 MHz), ^{13}C{^{1}H} (150 MHz) NMR spectra of **3m** in DMSO-d_6

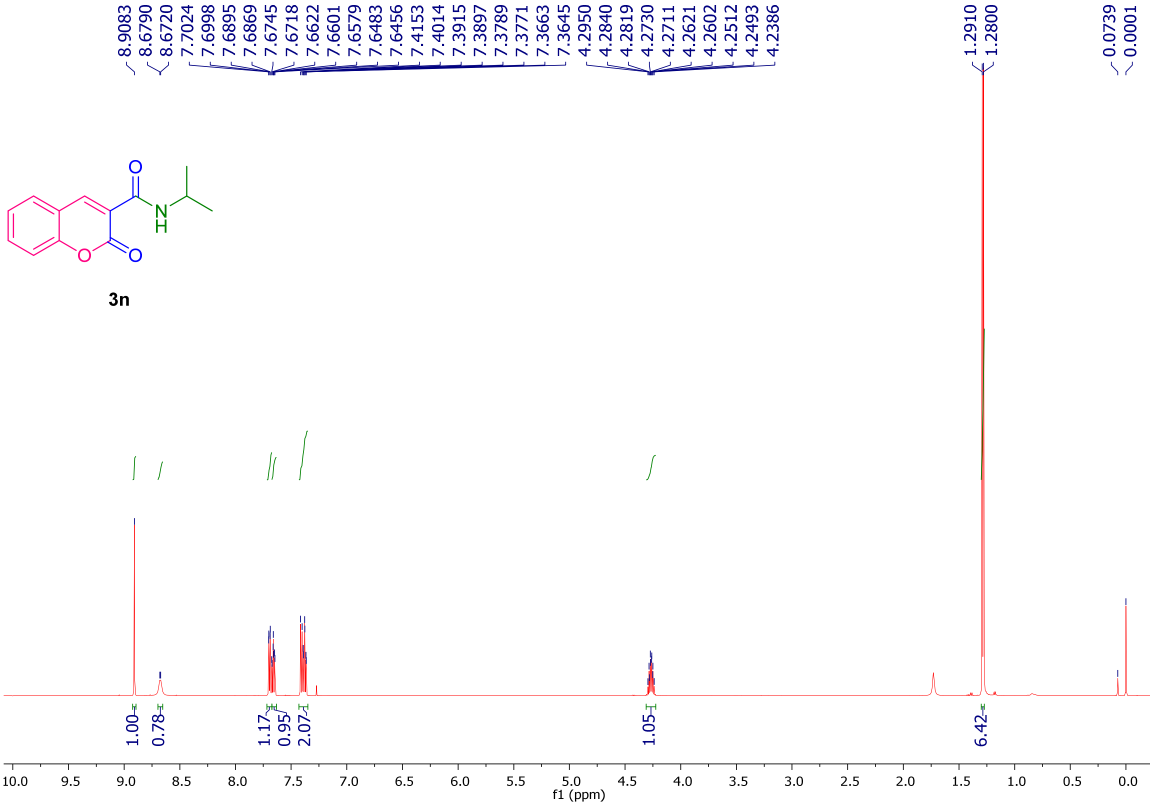

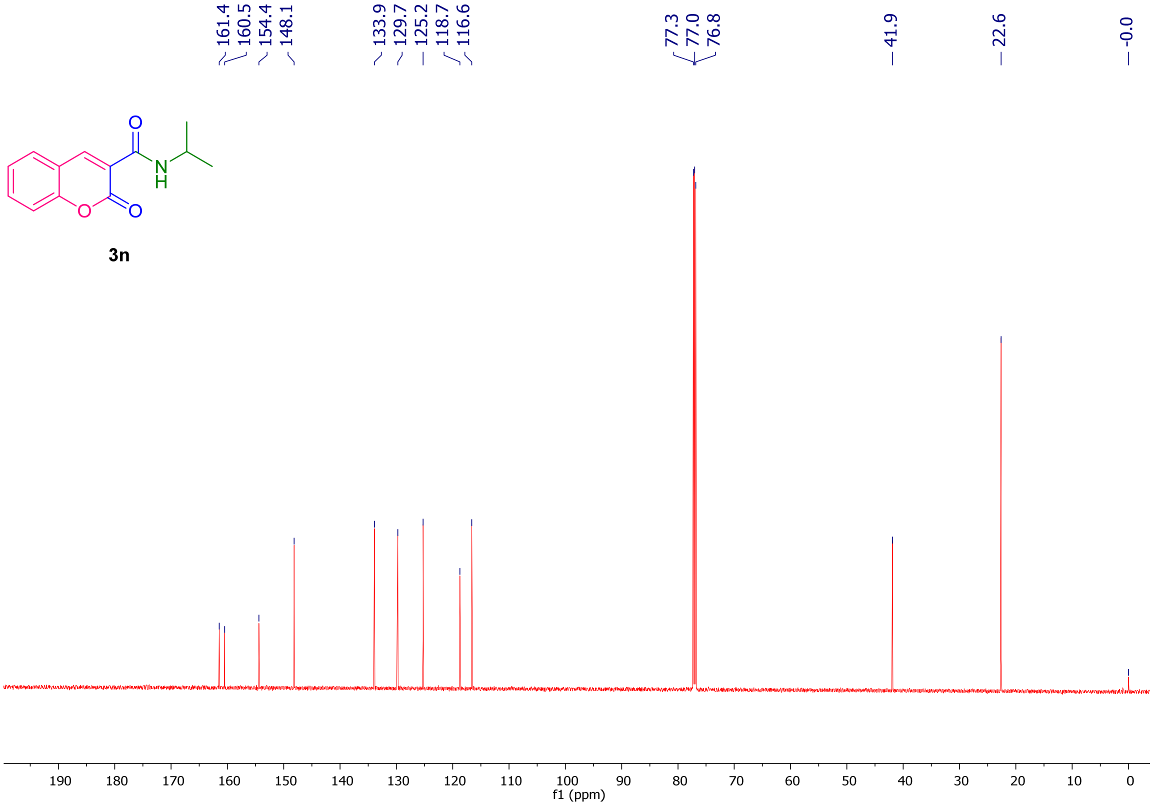

Figure S14. ^{1}H (600 MHz), ^{13}C{^{1}H} (150 MHz) NMR spectra of **3n** in $CDCl_3$

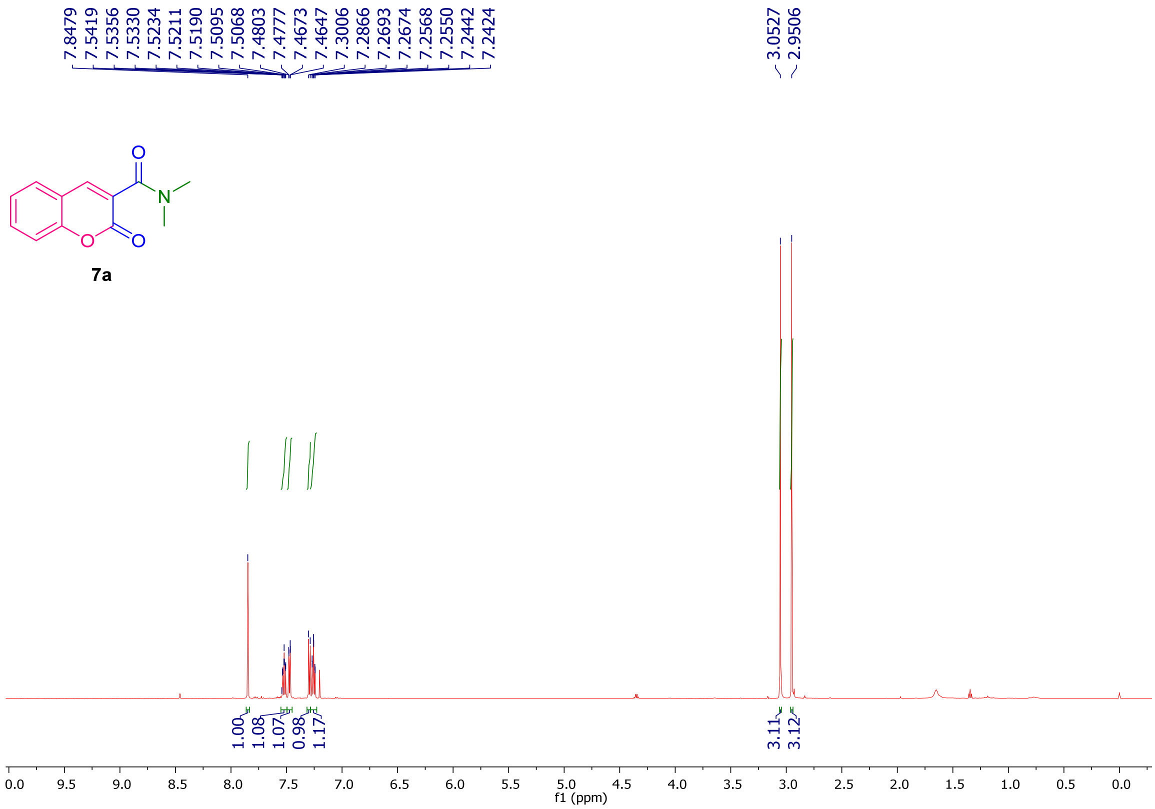

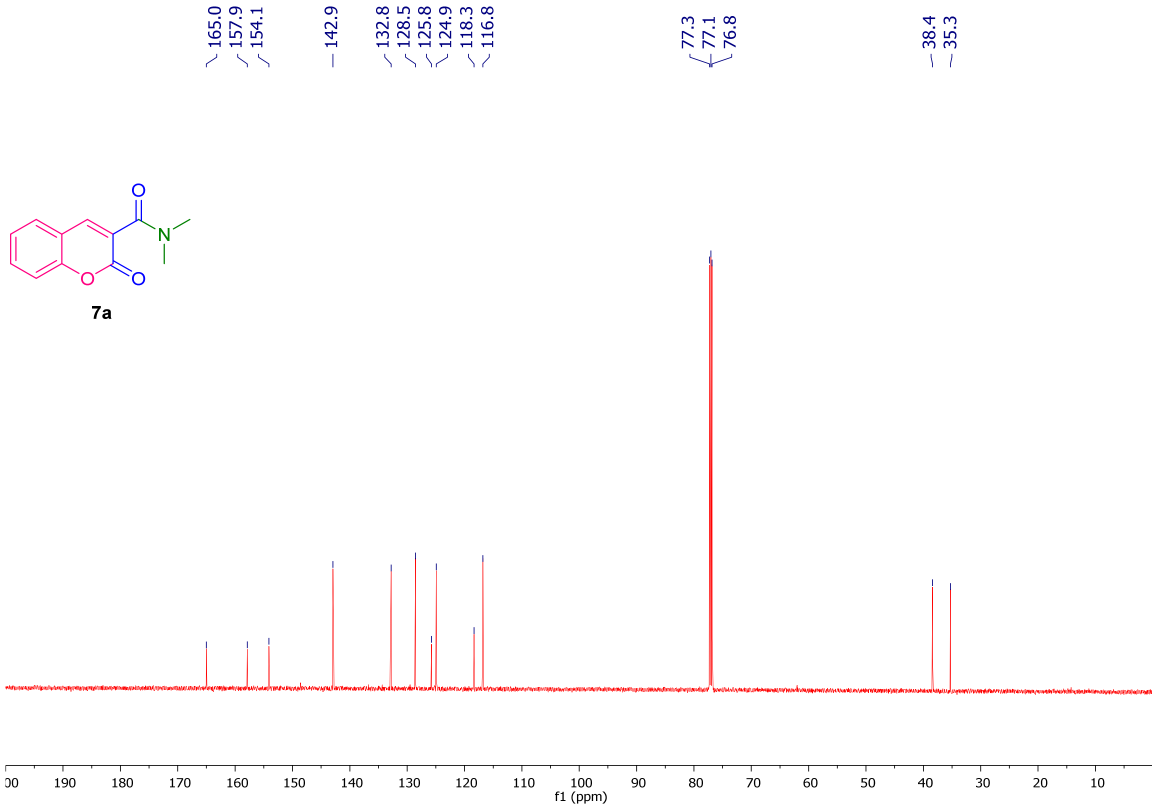

Figure S15. ^{1}H (600 MHz), ^{13}C{^{1}H} (150 MHz) NMR spectra of **7a** in $CDCl_3$

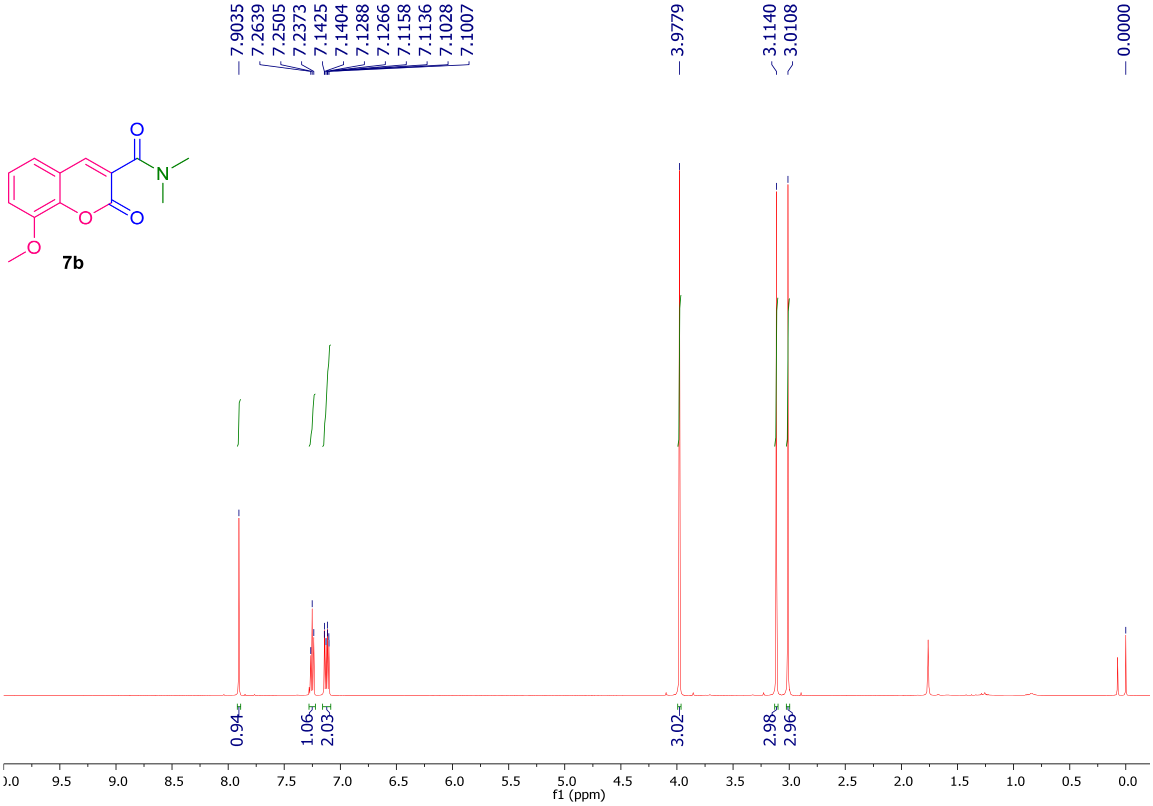

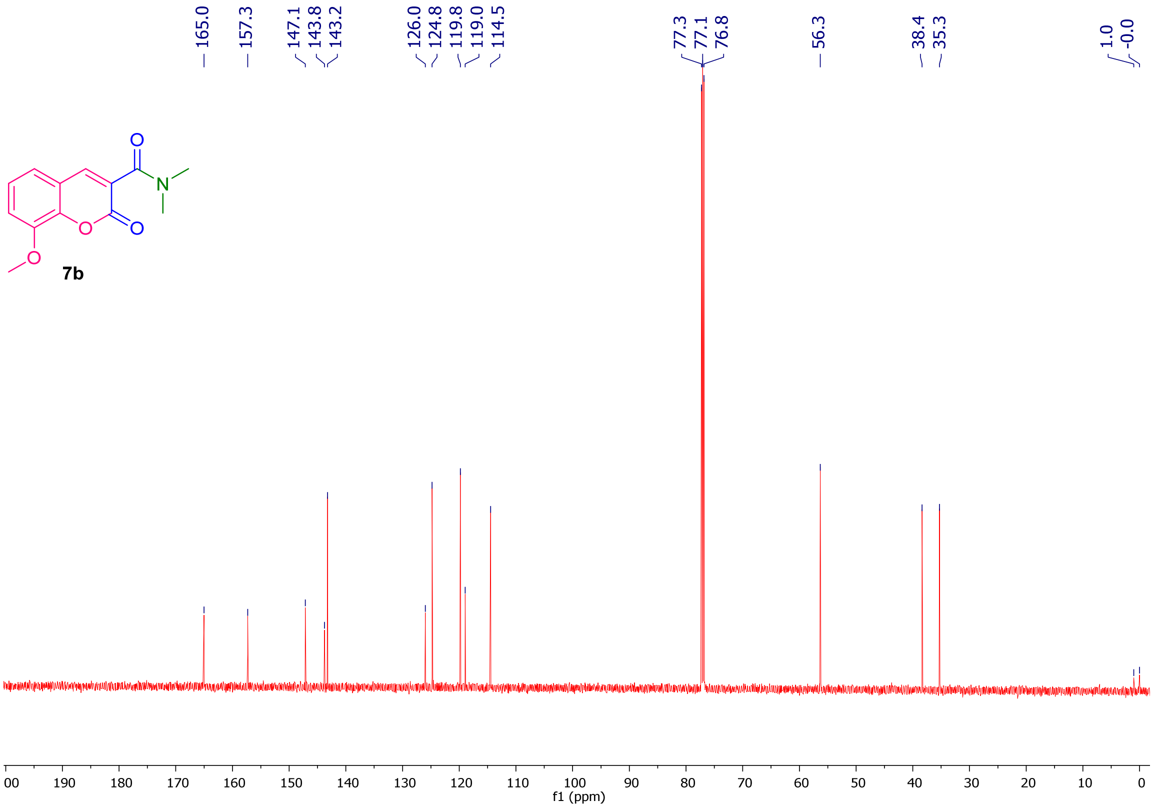

Figure S16. ^{1}H (600 MHz), ^{13}C{^{1}H} (150 MHz) NMR spectra of **7b** in $CDCl_3$

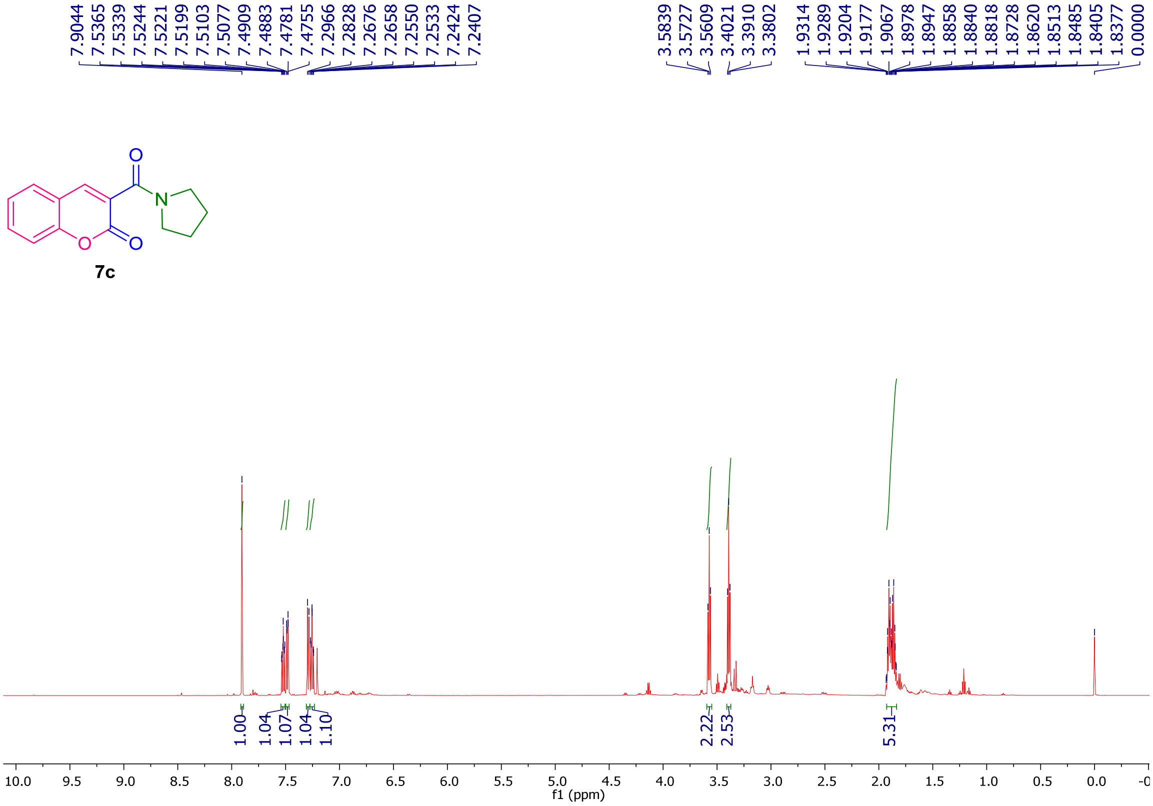

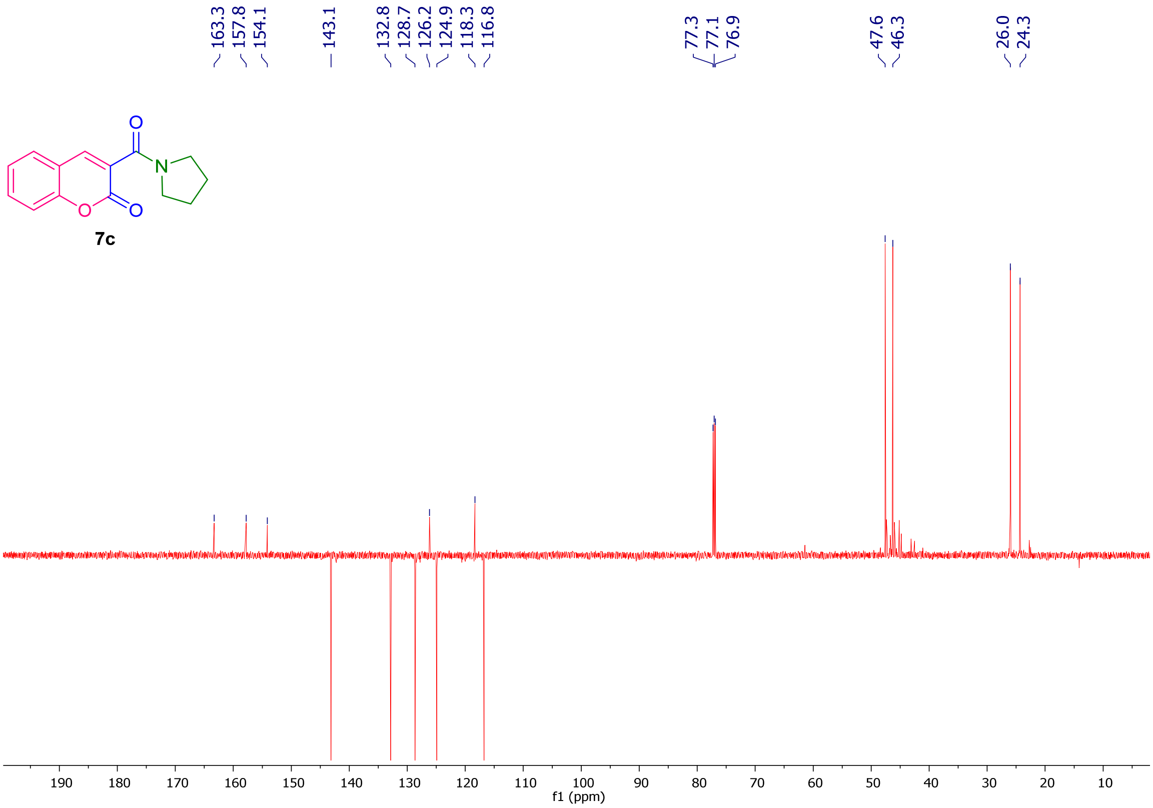

Figure S17. ^{1}H (600 MHz), DEPTQ {^{1}H} (150 MHz) NMR spectra of **7c** in $CDCl_3$

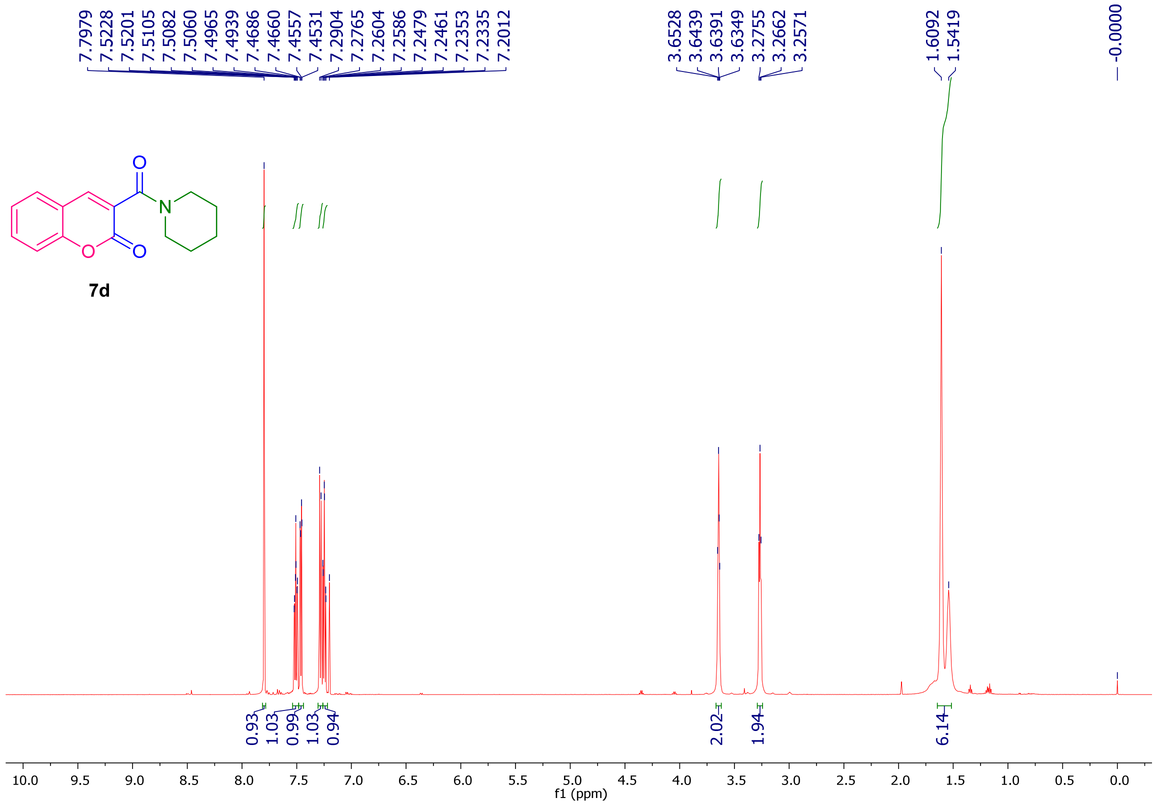

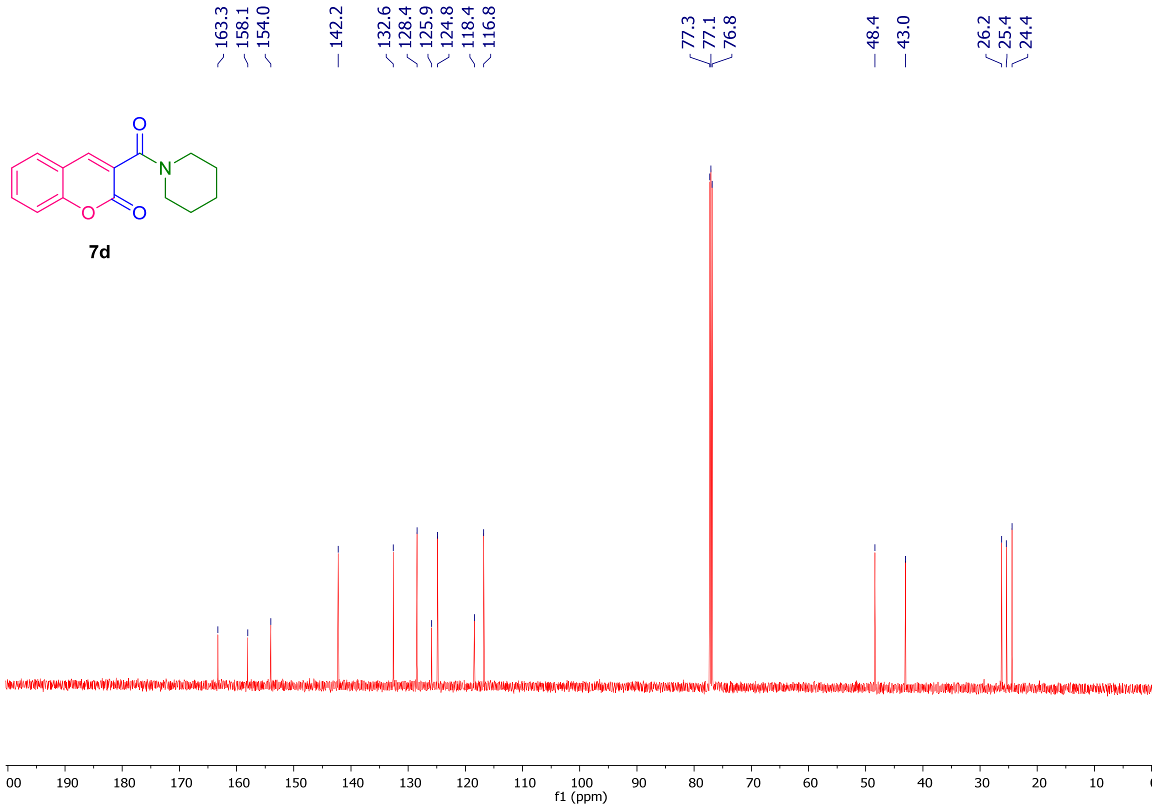

Figure S18. ¹H (600 MHz), ¹³C{¹H} (150 MHz) NMR spectra of **7d** in $CDCl_3$

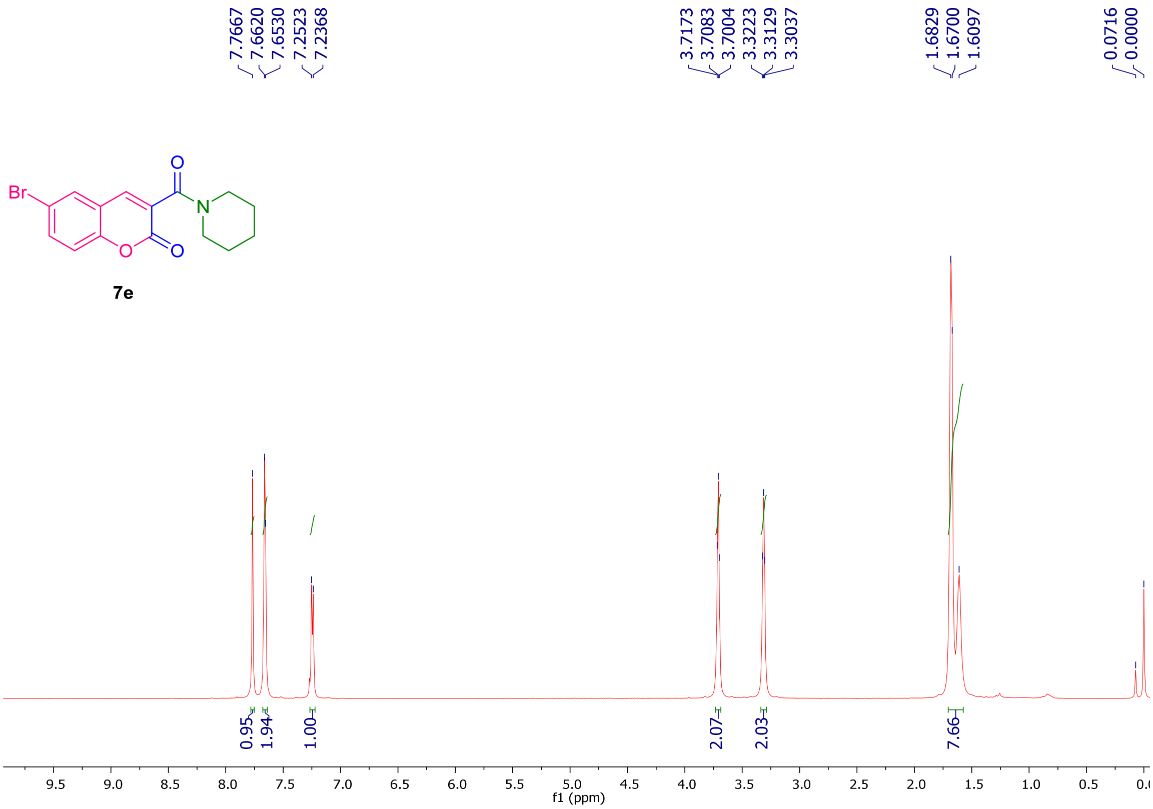

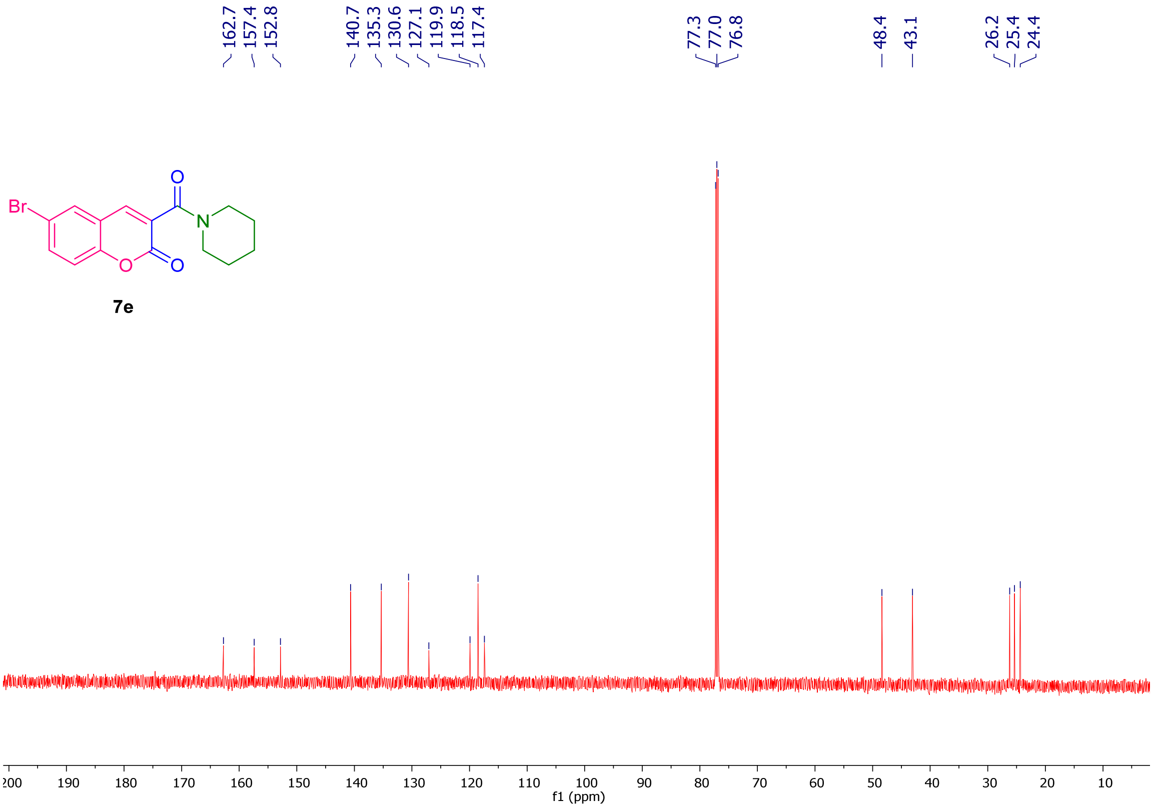

Figure S19. ^{1}H (600 MHz), $^{13}C\{^{1}H\}$ (150 MHz) NMR spectra of **7e** in $CDCl_3$

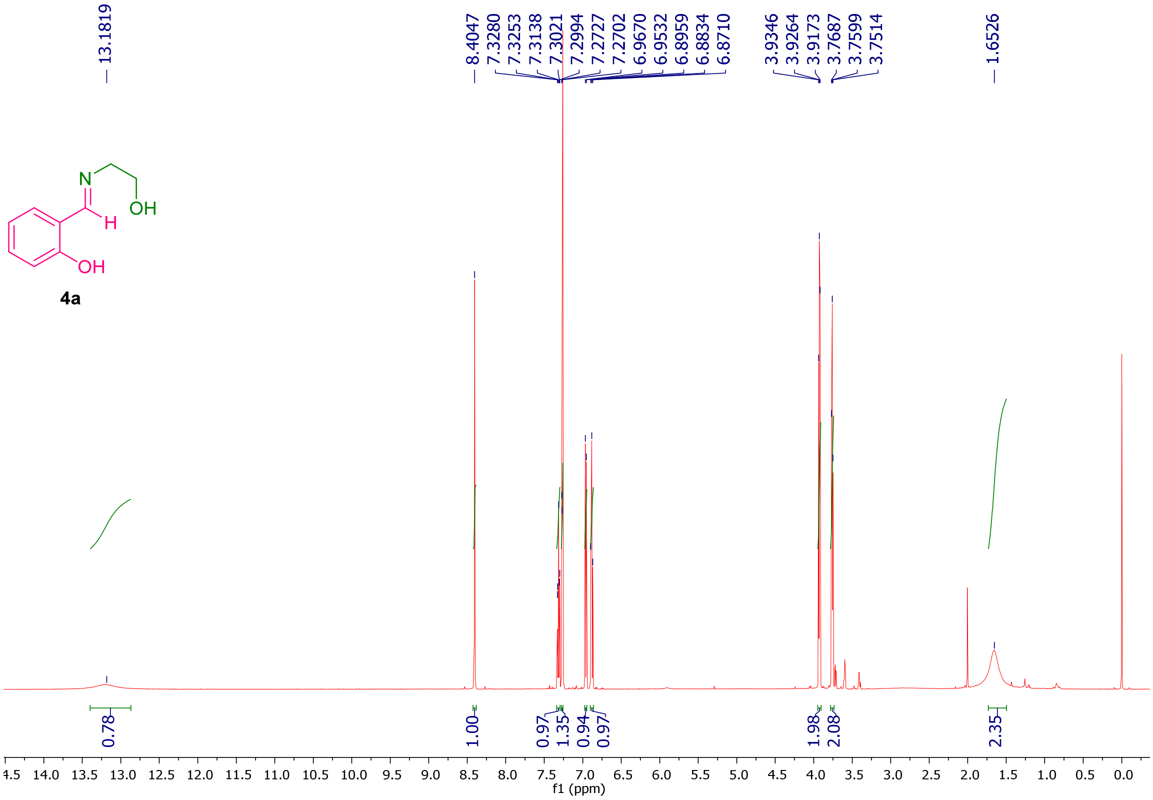

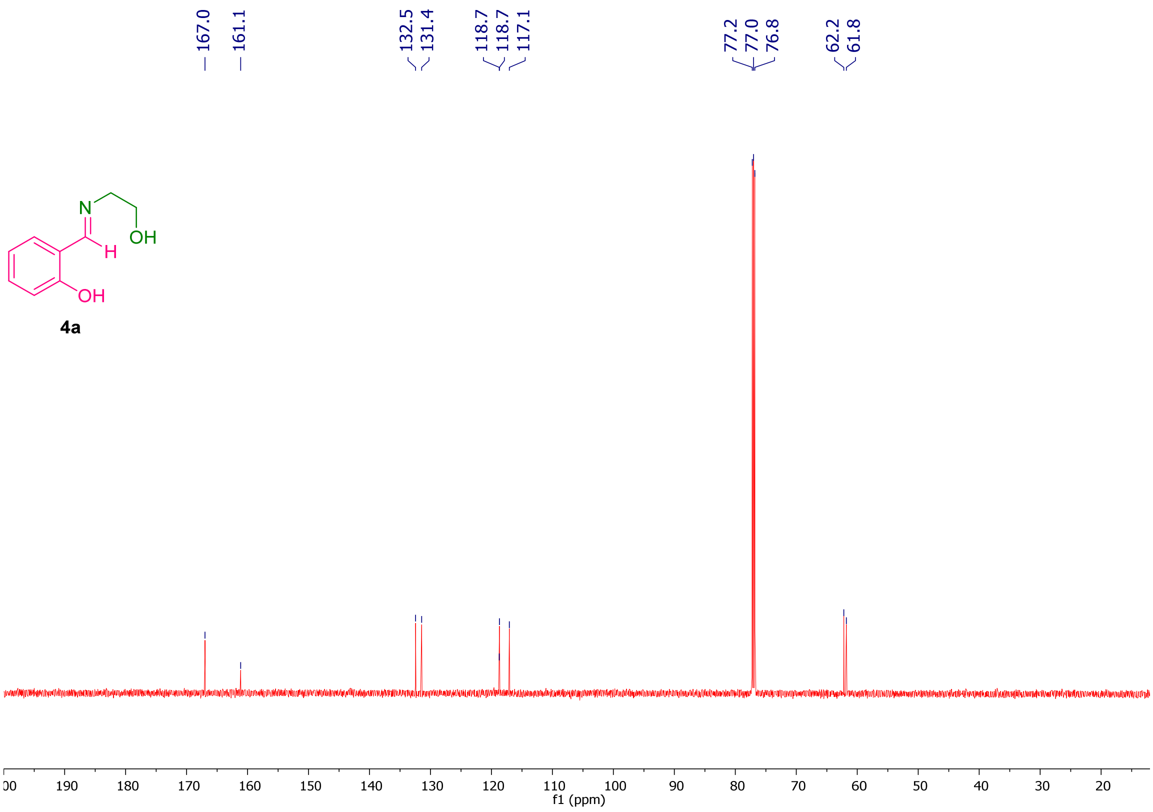

Figure S20. ^{1}H (600 MHz), $^{13}C\{^{1}H\}$ (150 MHz) NMR spectra of **4a** in $CDCl_3$

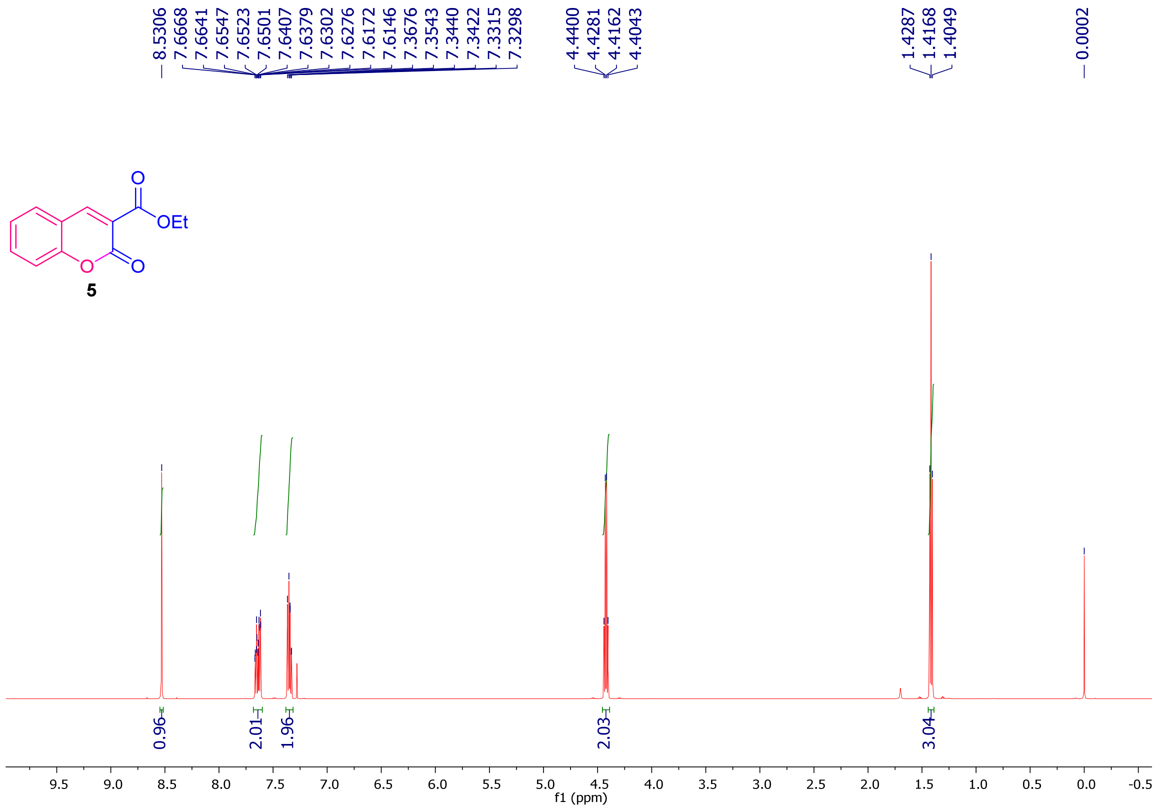

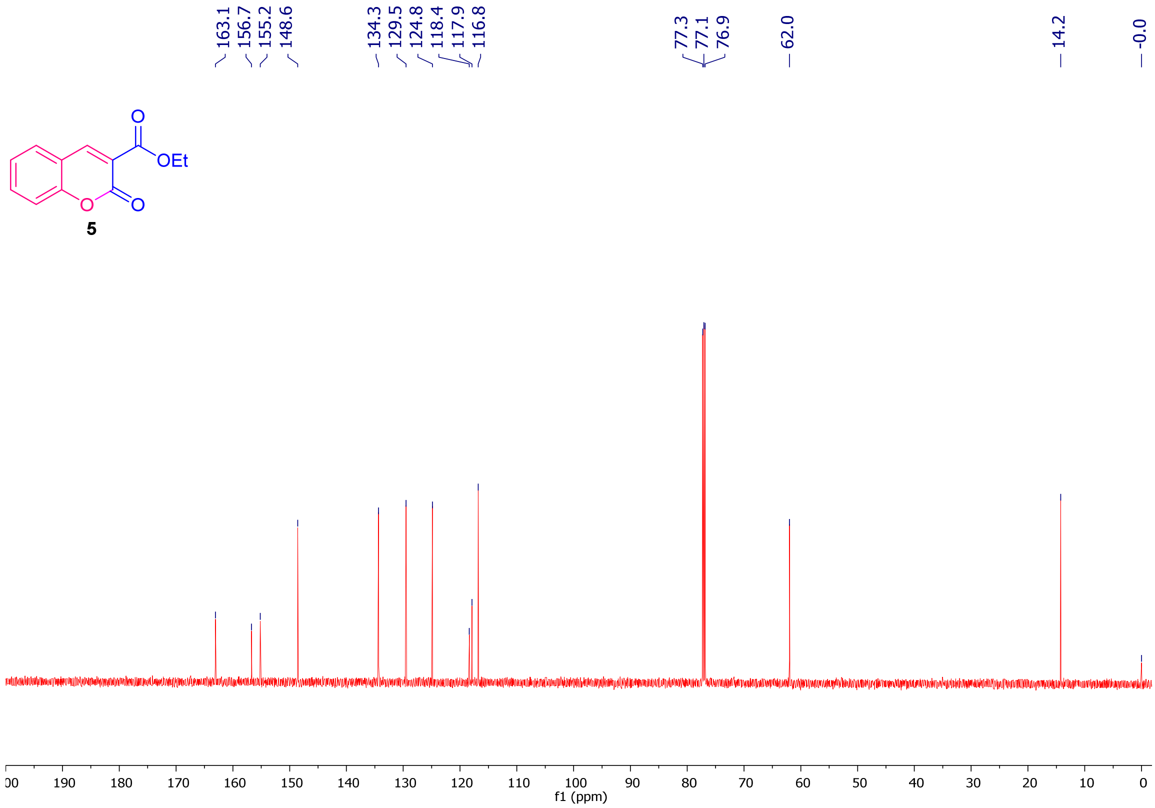

Figure S21. ^{1}H (600 MHz), $^{13}C\{^{1}H\}$ (150 MHz) NMR spectra of **5** in $CDCl_3$

Mechanistic details for the formation of **3c**

The differences of yields can be explained by the amines that participate in the multicomponent reaction: (*R*)-2-phenylglycinol (**3b**, 50%), (*S*)-methylbenzylamine (**3m**, 50%), isopropylamine (**3n**, 43%) and 2-amino-2-(hydroxymethyl)propane-1,3-diol (tris) (**3c**, 88%). Even though all four are bulky amines, tris has one advantage over the first three, it has three hydroxyl groups. As can be seen from the proposed mechanism, once the imine (**3c**) is formed between salicylaldehyde **1** and tris **2c**, the hydroxyl groups of tris can trap diethyl malonate via hydrogen bonds (two hydrogen bonds can be formed between tris and diethylmalonate) and then direct it directly to the electrophilic imine. Thus, the yield is increased. Besides, hydrogen bond also helps to activate the diethyl malonate carbonyls to the nucleophilic acyl substitution (Scheme S1).

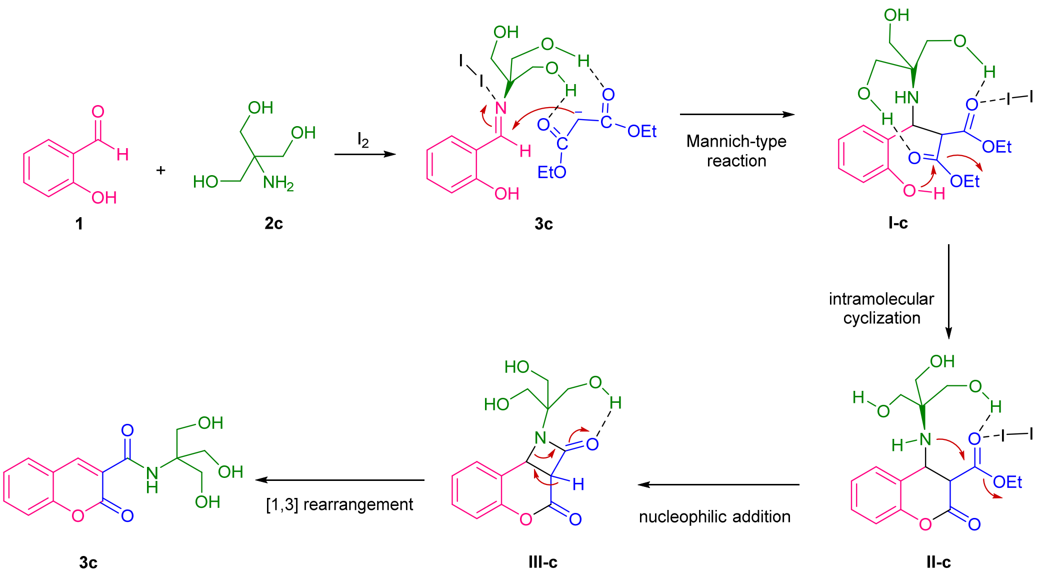

Scheme S1. Hydrogen bond helps to activate the diethyl malonate carbonyls to the nucleophilic acyl substitution.
